# Integrated physiological, transcriptomic, and metabolomic analyses elucidate the mechanism of salt tolerance in *Reaumuria soongorica* mediated by exogenous H₂S

**DOI:** 10.1186/s12870-025-07792-0

**Published:** 2025-12-01

**Authors:** Hanghang Liu, Xiaolan Li, Xidui Wang, De Zhang, Bingbing Tan, Ariunaa Ochir, Peifang Chong

**Affiliations:** 1 College of Forestry, Gansu Agricultural University, Lanzhou, 730070 China; 2 Mongolia Plant Protection Research Institute, Ulaanbaatar, 999097 Mongolia

**Keywords:** Reaumuria soongorica, Root, Hydrogen sulfide, Salt stress

## Abstract

**Background:**

Exogenous substances intervention constitutes a pivotal strategy for plant breeding and improvement, and is crucial for the remediation of saline-alkaline soils and the development of high-quality halophytic germplasm resources. Hydrogen sulfide (H₂S) is a novel gaseous signaling molecule that plays an important role in response to abiotic stress. For this purpose, this study integrates physiological and molecular biological research methods, focused on the cultivation of *Reaumuria soongorica* seedlings as the main research content, with *R. soongorica* roots as its main research object. This study aims to explore the regulatory effects of exogenous H₂S on *R. soongorica* at the seedling stage under salt stress and assess its potential to improve salt tolerance after seedling establishment.

**Results:**

Under salt stress, the activities of antioxidant enzymes in *R. soongorica* root increased, but high salt stress led to membrane lipid peroxidation and ion imbalance. Exogenous NaHS enhanced salt tolerance by enhancing antioxidant capacity, optimising osmolytes adjustment and maintaining ion homeostasis. This effect is particularly pronounced under 200 mM NaCl stress. Meanwhile, exogenous H₂S optimised carbon allocation toward root morphogenesis through the regulation of key carbon metabolism genes. It suppressed the energy-intensive proline OAT synthesis pathway while activating the “ALDH7A1–ADC” synergistic axis, thereby reducing energy expenditure while maintaining osmolyte balance. Concurrently, H₂S fine-tuned starch degradation and trehalose metabolism, thereby coordinating energy supply with osmolyte regulation. Collectively, these mechanisms improved growth and salt tolerance in *R. soongorica* root.

**Conclusions:**

Salt stress inhibited the root growth of *R. soongorica*. NaHS pretreatment enhanced antioxidant defense, regulated osmolytes balance, maintained ion homeostasis, and optimised energy allocation through a tripartite regulation mechanism involving proline-soluble sugars-starch. This effectively improved the salt tolerance of *R. soongorica* in the early growth stage. Therefore, it is crucial to develop preparations primarily composed of NaHS as a donor for seed pretreatment or seedling-stage application. Moreover, integrating H₂S treatment with existing agronomic practices such as slightly saline irrigation and soil conditioner application can form a low-cost, high-efficiency crop improvement technology for saline-alkali land is of great significance.

**Supplementary Information:**

The online version contains supplementary material available at 10.1186/s12870-025-07792-0.

## Introduction

Driven by global climate change and frequent human activities, the escalating severity of soil salinisation and alkalization has become a primary obstacle to local ecological and economic development [1]. In northwestern China, saline-alkaline soils are extensively distributed and characterised by severe salt accumulation, while the region also harbors abundant halophytic plant resources [2]. Although halophytes have an advantage over other plants for normal or better growth in saline-alkali soils [3], factors such as low rainfall, high evaporation rates, and improper irrigation methods in arid and semi-arid regions exacerbate the excessive accumulation of salt in the soil [2]. Notably, in saline–alkaline soils, capillary action drives pronounced surface salt accumulation, which severely threatens the initial germination and early growth of halophytic plants. This results in uneven distribution and sparse growth of salt-tolerant plants, limiting their role in saline-alkali land remediation [3]. As the cultivation of halophytes to improve saline-alkali land is currently one of the main strategies [4], the breeding of high-quality halophytes, the enhancement of their resistance, and their widespread cultivation have become crucial areas for future research.

*Reaumuria soongorica* is a salt-secreting perennial subshrub [5]. It is highly resistant to adverse conditions and ecologically adaptable, and its unique salt gland structure is essential to its adaptation to saline-alkali environments. It is primarily distributed in northwestern China’s inland regions (e.g., Xinjiang, Inner Mongolia, Qinghai) and serves as a dominant species in desert and grassland ecosystems [6]. It has strong salt tolerance, drought resistance, and water retention capabilities, and plays an active role in the ecological protection and construction of desert and grassland areas [5]. Simultaneously, *R. soongorica* communities also serve as valuable shrub forage in desert regions. However, the distribution of saline-alkaline soils remains one of the principal factors influencing their survival [7]. As a typical salt-secreting plant, *R. soongorica* preferentially absorbs Na^+^, and low concentrations of Na^+^ are conducive to its growth and development [8]. Studies have shown that the salinity threshold for *R. soongorica* seed germination is 200 mM NaCl [9, 10], but salinity concentrations in northwestern China’s saline soils often exceed this threshold [11, 12], resulting in severe inhibition of germination and early growth. Previous studies have shown that *R. soongorica* can tolerate up to 500 mM NaCl after seedling establishment [13]. This makes it a candidate for use as a model plant for saline-alkali land remediation. Nevertheless, biomass accumulation in *R. soongorica* from seed germination through seedling establishment is extremely slow; when salt stress is imposed during this period, its accumulation rate is further diminished. This is one of this species’ primary survival challenges. Although *R. soongorica* mitigates salt stress on aboveground tissues via leaf salt excretion and reactive oxygen species (ROS) regulation [14], subsurface soluble salts pose the primary threat during both seed germination and root development. Its resistance mechanisms against this stress remain relatively limited. Thus, developing effective strategies to improve *R. soongorica* seedling growth under adverse saline-alkaline conditions is critical.

Exogenous substance application is a core strategy for plant breeding and improvement, contributing significantly to saline-alkali land remediation, stress tolerance enhancement, and yield optimisation [15]. Its rapid responsiveness, ease of application, multi-target regulatory potential, low cost, and high efficacy offer clear advantages over conventional breeding and gene-editing approaches in terms of timeframe, expense, and applicability [16, 17]. Hydrogen sulfide (H₂S) is an inorganic compound analogous to carbon monoxide (CO) and nitric oxide (NO) [18]. As a signaling molecule in plants, it participates in regulating various physiological activities, promotes seed germination, enhances root development, and participates in plant salt tolerance resistance [19–21]. Numerous studies show that exogenous H₂S effectively alleviates salt stress-induced growth inhibition in plants [21, 22]. Low concentrations (0.05–0.10 mmol L⁻¹) of H₂S significantly increase seed germination rates and seedling biomass in *Nicotiana tabacum* L [23]. Exogenous H₂S treatment can also significantly increase plant biomass and enhance overall salt tolerance [24, 25]. H₂S treatment at 0.05 mmol·L⁻¹ can significantly alleviate the growth inhibition of plant roots caused by salt stress [23, 26]. Additionally, hydrogen sulfide is a small, lipophilic molecule that readily crosses cell membranes and modulates diverse functions [27]. The mechanism of action of hydrogen sulfide primarily involves extensive post-translational regulation through disulfide modification [28]. It integrates multiple signaling pathways to regulate redox and ionic homeostasis in plants under salt stress, thereby mitigating oxidative damage and enhancing salt tolerance [29]. Correlative omics analysis revealed that under salt stress, NaHS alleviates oxidative damage in rice seedlings by upregulating the expression levels of proteins associated with ATP synthesis [30]. Concurrently, H₂S promoted the binding of Plasma membrane (*PMA1*) and GENERAL REGULATORY FACTOR 4 (*GRF4*) in *Arabidopsis* through a transulfuration mechanism. This activates *PMA* and maintains K⁺/Na⁺ homeostasis and salt tolerance under salt stress [31]. H₂S also mediates enhanced binding of precursor mRNAs for the salt stress response genes Cysteine-rich Receptor-like Protein Kinase 42 (*CRK42*) and Vascular Plant One Zinc Finger Protein (*VOZ1*) in *Arabidopsis*, thereby enhancing salt tolerance [32]. Also, a low concentration of NaHS significantly alleviates the growth inhibition of barley caused by 150 mM NaCl. It modulates the K^+^/Na^+^ balance by decreased K⁺ efflux and increased expression levels of the inward-rectifying potassium channel (*HvAKT1*), thereby enhancing salt tolerance by maintaining ionic homeostasis [33]. Furthermore, H₂S acts as a novel inducer of lateral root formation by stimulating the expression of cell cycle regulatory genes (*CCRGs*) [34], and is recognised as one of the key regulatory factors in plant responses to salt stress [31, 35]. This effect was also observed in the regulation of lateral root formation in *Prunus persica*. The expression levels of SUCROSE NON-FERMENTING RELATED KINASE 1 (*PpSnRK1*) in *Prunus persica* roots increased under hydrogen sulfide treatment, thereby promoting lateral root development [36]. In biological research, sodium hydrosulfide (NaHS) is commonly used as an exogenous H₂S donor. NaHS dissociates in vivo into Na⁺ and HS⁻, which can then combine with H⁺ to form H₂S, thereby establishing a dynamic equilibrium that enables the application of exogenous H₂S [37].

In recent years, as research into the use of small-molecule compounds to regulate plant salt-tolerance mechanisms has expanded, H₂S has emerged as a novel small-molecule regulator whose roles in modulating plant physiological and metabolic responses under stress have increasingly become a focal point. Previous studies have demonstrated that treatment with 0.025 mM NaHS can alleviate the effects of salt stress on the growth and development of *R. soongorica* seedlings [38]. However, whether exogenous H₂S application can similarly alleviate salt-induced growth inhibition during the seedling stage of *R. soongorica* remains to be elucidated. We hypothesise that exogenous H₂S can effectively alleviate the inhibitory effects of salt stress on the growth of *R. soongorica* and improve its salt tolerance during the early growth stage by regulating its physiological and molecular metabolic pathways. For this purpose, this study focused on the cultivation of *R. soongorica* seedlings as the main research content, with *R. soongorica* roots as its main research object. The aim was to elucidate the regulatory effects of exogenous H₂S on *R. soongorica* during the seedling stage under salt stress and to assess its potential to enhance salt tolerance following seedling establishment. Integrate physiological and molecular biological research methods to conduct more in-depth research into its regulatory mechanisms. Exploring strategies to improve the survival and reproduction rates of *R. soongorica* seedlings in saline-alkali desert habitats is crucial for developing high-quality germplasm resources and their application in ecological restoration.

## Materials and methods

### *R. soongorica* seedling cultivation

The experiment utilised seedling trays with 50 holes, characterised by a total length and width of 54 cm and 28 cm, respectively. The diameter at the top and bottom was measured at 4.8 cm and 1.5 cm, respectively, with a height of 11 cm. Initially, the trays must be filled with the experimental substrate, which consists of loam: peat soil: fine sand in a ratio of 3:1:1. A total of 10 seedling trays were divided into two groups for the administration of solution (NaCl + H2O, NaCl + NaHS), and each group was soaked in 0, 50, 100, 200, and 300 mM NaCl solution for 10 min. Following the saturation of the experimental substrate in the seedling trays, the trays were removed and permitted to drain fully. The resulting media were designated as the experimental salt-stressed substrates.

**Fig. 1:**
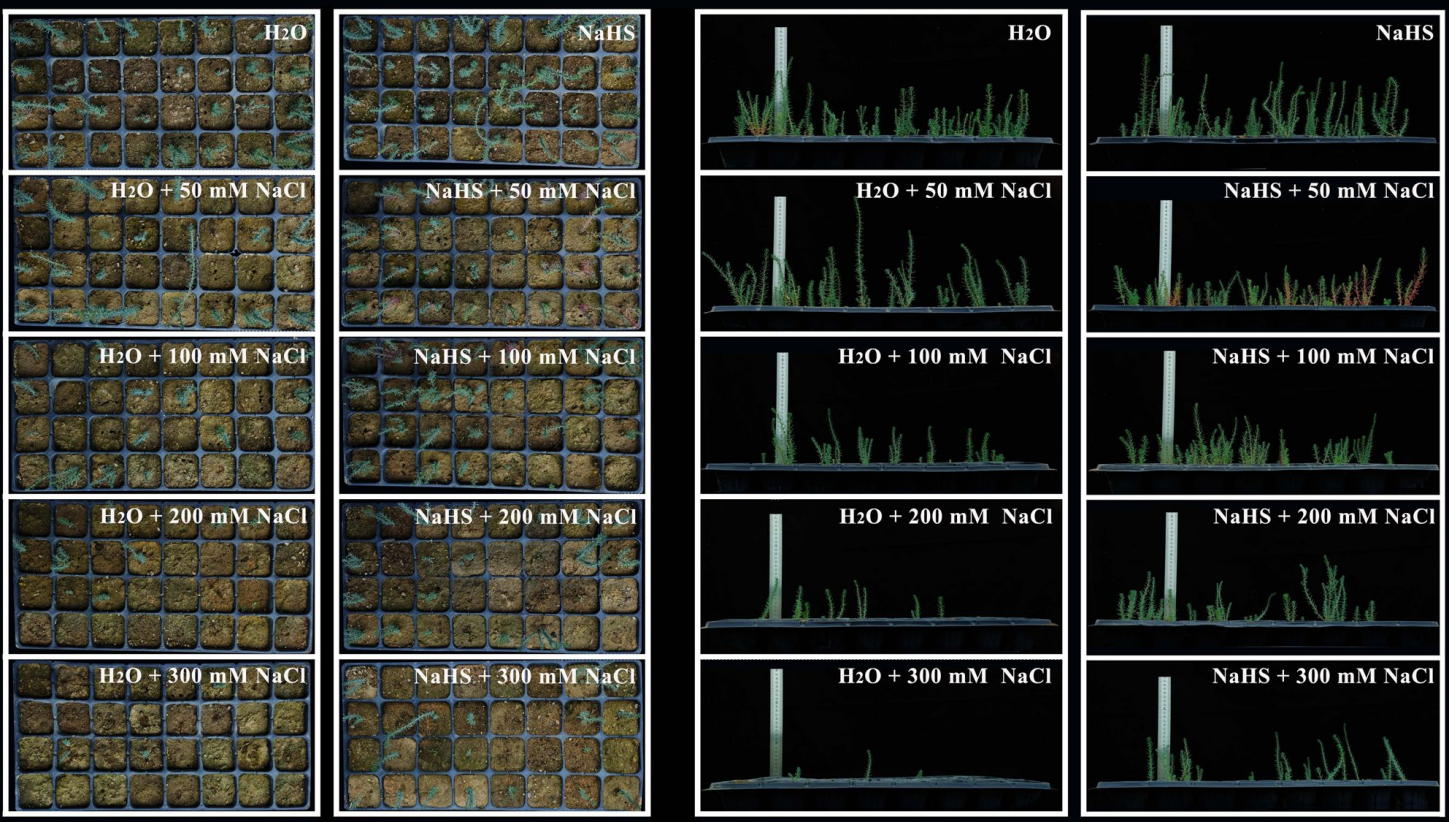
Effect of exogenous H₂S on *R. soongorica* trays-seedling cultivation under salt stress

The *R. soongorica* seeds utilised in the present experiment were collected from the Gansu Wuwei City Seed Breeding Center (38°24′ N, 103°9′ E) in Yangxiaba Town, Liangzhou District, Wuwei City, Gansu Province. Seeds of uniform size were selected for this purpose. The instruments were then subjected to a process of disinfection, involving the application of 75% alcohol for a duration of 3 min. This was followed by a rinsing procedure with distilled water, which was repeated a total of 4 times. The instruments were then dried using absorbent paper. *R. soongorica* seeds were then sown into the seedling trays, with four seeds placed in each hole. The two treatment groups of the seedling trays were positioned under an artificial rain shelter at Gansu Agricultural University’s research base. The commencement of the experiment was scheduled for April 1, 2024. The single-salt treatment group (NaCl + H₂O) consisted of five seedling trays that were irrigated with groundwater. The following concentrations were established: 0 mM NaCl + H₂O (W), 50 mM NaCl + H₂O (WS50), 100 mM NaCl + H₂O (WS100), 200 mM NaCl + H₂O (WS200), and 300 mM NaCl + H₂O (WS300). The NaCl + NaHS treatment group comprised five seedling trays that were irrigated with a 0.025 mM NaHS solution. The following concentrations were established: 0 mM NaCl + NaHS (H), 50 mM NaCl + NaHS (HS50), 100 mM NaCl + NaHS (HS100), 200 mM NaCl + NaHS (HS200), and 300 mM NaCl + NaHS (HS300). The details of the treatment are shown in Table S1. For both treatment groups, 5 mL of the corresponding solution was injected into each hole of the seedling trays using a continuous syringe at 7 a.m. and 7 p.m. each day (5 mL of solution is sufficient to submerge the *R. soongorica* seeds and prevent the loss of NaCl). The *R. soongorica* during the seedling stage were subject to daily monitoring, with treatments being applied continuously for a period of four months (Fig. 1). At the end of the treatment period, all seedlings from each group were fully sampled. Roots were meticulously rinsed with distilled water, rapidly frozen in liquid nitrogen, and subsequently stored in an ultra-low temperature freezer set at − 80 °C. This ensured the preservation of physiological and biochemical indicators, transcriptomic and metabolomic analyses.

### Determination of growth parameters

Three *R. soongorica* seedlings were randomly selected, rinsed thoroughly with distilled water, and dried with absorbent paper. The seedlings were separated at the root-stem junction, and fresh root weight was recorded. Root morphological parameters, including surface area and total length, were measured using root analysis software (WinRHIZO 2008a). The roots were then oven-dried at 105 °C for 15 min, followed by drying at 75 °C until a constant weight was reached. The dry root weight was then determined. To investigate changes in the growth of *R. soongorica* plant height, three seedlings were randomly selected and their growth measured over a period of 30 days. The plant height was determined by measuring the distance from the base of the plant to the top of the main stem with a ruler.

### Determination of MDA and proline

The concentration of malondialdehyde (MDA) was determined using the method [39]. A 0.2 g root sample was placed in a mortar cooled in an ice bath, ground to a homogeneous slurry with a small amount of quartz sand and 2 mL of 10% (w/v) trichloroacetic acid (TCA), and the mortar was then rinsed three times with a total of 3 mL of 10% TCA. The combined extract was transferred to a centrifuge tube and brought to 10 mL with 10% TCA, then centrifuged at 4 °C, 4,000 × g for 10 min. A 2 mL aliquot of the supernatant was withdrawn and mixed with 2 mL of 0.6% (w/v) thiobarbituric acid (TBA) solution. The reaction mixture was incubated in a boiling water bath for 15 min, timing beginning upon the first appearance of bubbles in the tube. Upon completion, tubes were immediately immersed in an ice-water bath to cool rapidly, and the mixture was centrifuged again under the same conditions. Absorbance was recorded at 532, 600, and 450 nm, and MDA content was calculated.

Proline content was determined spectrophotometrically following the method [40]. A 0.2 g root sample was placed in a mortar chilled in an ice bath and ground to a homogeneous slurry with a small amount of quartz sand and 3% (w/v) sulfosalicylic acid. The slurry was transferred to a 10 mL centrifuge tube and brought to 5 mL with 3% (w/v) sulfosalicylic acid. The suspension was incubated in a boiling water bath for 10 min, cooled to room temperature, and centrifuged at 25 °C, 4,000 × g for 10 min. A 2 mL aliquot of the supernatant was transferred to a test tube, to which 2 mL of glacial acetic acid and 2 mL of 2.5% (w/v) acidic ninhydrin solution were added. The reaction mixture was then heated in a boiling water bath for 30 min. After cooling, 4 mL of toluene was added in a fume hood, the mixture was shaken vigorously, and the phases were allowed to separate over 3 h. The toluene phase was collected, and its absorbance was read at 520 nm. Proline concentration was calculated from a standard curve (0–20 µg/mL).

### Determination of soluble protein, soluble sugar, and starch content

Soluble protein content was determined by the Coomassie Brilliant Blue G-250 assay [40]. A 0.2 g root sample was placed in an ice-cold mortar, ground with a small amount of quartz sand and 5 mL of extraction buffer (5 mM EDTA, 0.5% PVP, 50 mM phosphate buffer, pH 7.8) to yield a homogeneous slurry, transferred to a centrifuge tube, and adjusted to 8 mL with the same buffer. The extract was centrifuged at 4 °C, 12,000 × g for 10 min. Then, 0.5 mL of the supernatant was mixed with 0.5 mL of distilled water and 5 mL of Coomassie Brilliant Blue G-250. After shaking well and leaving for 2 min, the absorbance at 595 nm was recorded (the control was 1 mL of distilled water + 5 mL of Coomassie Brilliant Blue G-250). Soluble protein concentration was calculated from a bovine serum albumin standard curve (0–100 µg/mL).

Soluble sugar content was determined by the anthrone-sulfuric acid method [41]. A 0.2 g root sample was ground with quartz sand, transferred into a centrifuge tube, and extracted with 10 mL of distilled water in a boiling water bath for 30 min. After cooling to 25 °C and centrifugation at 25 °C, 4,000 × g for 10 min, the supernatant was collected. The residue was re-extracted twice with 10 mL of distilled water under the same conditions, and the combined extracts were adjusted to 100 mL. An aliquot of 1 mL extract was mixed with 1 mL distilled water and 0.5 mL anthrone reagent, followed by the slow addition of 5 mL concentrated H₂SO₄. Cover the test tube with a stopper and shake gently, then place in a boiling water bath for 10 min (for the colorimetric blank, use 2 mL of distilled water mixed with 0.5 mL of anthrone reagent and place them together in a boiling water bath for 10 min). After cooling to room temperature, absorbance was measured at 620 nm, and soluble sugar content was calculated against a glucose standard curve (0–100 µg/mL).

Starch content was determined by the perchloric acid extraction method [41]. The residue remaining after soluble sugar extraction was transferred into a 50 mL volumetric flask, and 20 mL of hot distilled water was added. The mixture was boiled in a water bath for 15 min, followed by the addition of 2 mL of 9.2 mol L⁻¹ perchloric acid and a further 15 min extraction. After cooling, the suspension was filtered through filter paper, and the residue was subjected to two additional extractions with 10 mL of distilled water under the same conditions. The combined filtrates were collected as the starch extract. An aliquot of 0.5 mL of extract was mixed with 1.5 mL of distilled water and 0.5 mL of anthrone reagent, after which 5 mL of concentrated H₂SO₄ was added dropwise. After vigorous mixing, the tubes were immediately immersed in a boiling water bath and held at a temperature for precisely 1 min each (the colorimetric blank was prepared by mixing 2 mL of distilled water with 0.5 mL of anthrone reagent, and the mixture was then heated in a boiling water bath for 1 min). The optical density was measured at 620 nm after removal and cooling to room temperature.

### Antioxidant enzyme activities determination

Briefly, 0.2 g of root tissue was homogenised in 8 mL of extraction buffer (5 mM EDTA, 0.5% PVP, 50 mM phosphate buffer, pH 7.8). The homogenate was centrifuged at 4 °C, 12,000 × g for 15 min, and the resulting supernatant was used for the enzyme assays.

Superoxide dismutase (SOD) activity was determined by the spectrophotometric method, which measures the inhibition of the photochemical reduction of nitroblue tetrazolium (NBT) at 560 nm [39]. The 3 mL reaction mixture was composed of 0.1 mL of enzyme extract (0.1 mL of distilled water for the control), 1.5 mL of 50 mM phosphate buffer (pH 7.8), 0.3 mL of 0.1 mM ethylenediaminetetraacetic acid (EDTA), 0.3 mL of 130 mM L-methionine, 0.3 mL of 0.75 mM NBT, 0.3 mL of 0.02 mM riboflavin, and 0.5 mL of distilled water. Tubes were illuminated under cool white light at 5,000 lx for 10 min. Non-illuminated reactions and illuminated reactions lacking enzyme extract were used as calibration standards. One unit of superoxide dismutase activity was defined as the amount of enzyme required to achieve 50% inhibition of NBT reduction.

Catalase (CAT) activity was determined by monitoring the decomposition of H₂O₂ at 240 nm [39]. The 3.0 mL reaction mixture comprised 0.2 mL of enzyme extract and 2.8 mL of 25% (v/v) H₂O₂ in 100 mM phosphate buffer (pH 7.0) containing 0.1 µM EDTA. The decrease in absorbance at 240 nm was recorded over 2 min, and one unit of CAT activity was defined as the amount of enzyme extract causing an absorbance decline of 0.01 per minute.

Peroxidase (POD) activity was determined by measuring the H₂O₂-dependent oxidation of guaiacol [39]. One milliliter of enzyme extract was added to a reaction mixture comprising 1.5 mL of guaiacol solution and 0.5 mL of H₂O₂ solution. The increase in absorbance at 470 nm was recorded over 2 min, and one unit of POD activity was defined as the amount of enzyme causing a 0.01 absorbance increase per minute.

### Determination of the Na^+^ and K^+^ content

The flame photometry method was used to determine ion contents [42]. Root samples had been rinsed three times with deionized water to remove surface-adsorbed ions and then blotted dry with absorbent paper to prevent ion leaching. Samples were subsequently inactivated by heating at 105 °C for 30 min and oven-dried at 80 °C to constant weight. The dried roots were ground to a fine powder through a 60-mesh sieve. An aliquot of 0.2 g of the powdered root material was subjected to microwave digestion with 10 mL of concentrated HNO₃ until the solution had become transparent. After cooling, the digest was diluted to 100 mL with deionized water. The resulting solution was introduced into a flame atomic emission spectrometer (Shanghai Jingke FP640) to measure the emission intensities of Na⁺ and K⁺, and ion concentrations were calculated from calibration curves.

### Determination of endogenous H_2_S content

Endogenous H_2_S content was determined using the method [43]. Briefly, 0.2 g of root tissue was homogenised in 7.0 mL of extraction buffer (0.25% w/v zinc acetate, 10 mM EDTA, 100 mM phosphate buffer, pH 8.0) and centrifuged at 4 °C, 12,000 × g for 15 min. An aliquot of 3 mL of the supernatant was then mixed with 1 mL of 5 mM N, N-dimethyl-p-phenylenediamine and gently agitated for 12 min. Subsequently, 1 mL of 150 mM FeCl₃ was added, and the reaction was allowed to proceed for 15 min. The absorbance of the resulting solution was measured at 667 nm.

### Comprehensive evaluation of exogenous H₂S on the *R. soongorica* roots under salt stress

Each physiological parameter was assigned a defined role in plant stress responses. Under salt stress, the selected metrics were classified as positive or negative indicators and were subsequently standardized. Positive indicators, for which higher values denote stronger stress effects, were normalized by (treatment value/control value) × 100. Negative indicators, for which higher values denote weaker stress effects, were normalized by 1/(treatment value/control value) × 100. The standardized dataset was subjected to principal component analysis (PCA); principal components were extracted, and their variance contribution ratios were used to assign weights. The principal component scores were then calculated for each treatment group and normalized to the 0–1 range using the affiliation function. Composite scores were calculated and ranked according to the weights.

(1) Weighting formula [44]:

W_j_ = PCj/(PC1 + PC2 + … + PC_j_) (j = 1, 2, 3, …, n).

W_j_ is the weight of the j-th principal component; PC_j_ is the variance contribution rate corresponding to the j-th principal component (cumulative contribution rate ≥ 85%).

(2) Principal component score formula [45]:

F_j_ = Z_1_ × _1_+ Z_2_ × _2_ + … + Z_j_X_j_ (j = 1, 2, 3, *…*, n).

Where F_j_ is the principal component score; Z_j_ is the standardized variable; X_j_ is the score coefficient corresponding to the j-th principal component.

(3) Standardized calculation formula for the affiliation function [46]:

U_j_ =(X_j_-X_min_)/(X_max_-X_min_) (j = 1, 2, 3, …, n).

Where U_j_ is the principal component score after standardization of the affiliation function; X_j_ is the j-th principal component score; X_min_ is the minimum value of the j-th principal component score; X_max_ is the maximum value of the j-th principal component score.

(4) Principal component composite score formula:

CS_j_ = W_1_U_1_ + W_2_U_2_ + … + W_j_U_j_ (j = 1, 2, 3, *…*, n).

In this equation, the sum of the products of the weights (W_j_) of each j principal component and the principal component scores (U_j_) standardized by the corresponding affiliation functions, that is, the principal component composite score (CS_j_).

### RNA sample Preparation and transcriptomic analysis

Briefly, total RNA was extracted from the collected 50 mg of *R. soongorica* roots using the plant RNA purification kit (Norgen, Thorold, ON, Canada) according to the manufacturer’s instructions. RNA integrity was verified by 1% agarose gel electrophoresis, and RNA concentration and purity were assessed using a NanoDrop 2000 spectrophotometer (Thermo Fisher Scientific, Waltham, MA, USA). The obtained high-quality RNA (OD260/280 = 1.85 ~ 2.20, concentration ≥ 30 ng/µL) was finally used for library construction. The library preparation kit (BGI, Shenzhen, China) was used to enrich the mRNA with oligo(dT) (Biomag, Wuxi, China) magnetic beads and fragment it. Double-stranded cDNA was then synthesised via reverse transcription (with dUTP incorporation) to construct strand-specific libraries. The libraries were then amplified by PCR, circularised and processed into DNA nanospheres (DNBs). Sequencing was performed using combined probe-anchored polymerisation (cPAS) technology on the T10 sequencer (BGI, Shenzhen, China). First, we filtered out low-quality reads, reads with adapter contamination, and reads with excessively high levels of unknown bases (N). The filtered data are referred to as clean reads. In this step, clean reads were obtained by removing adapter sequences, reads containing more than 10% unknown bases and low-quality sequences from raw data. The resulting clean reads were assembled into unigenes using Trinity software (v2.13.2) after Q20, Q30, GC-content and sequence duplication level of the clean reads were calculated. All the downstream analyses were based on the high-quality clean reads. Fragments per kilobase of transcript per million fragments mapped (FPKM) were used to evaluate the expression level of genes or transcripts. DESeq2 was utilized for gene differential expression analysis among samples. At the same time, to obtain multiple differentially expressed genes, the screening criteria are an expression fold change |fold change| >1.42 and adjusted p-value (Q value) ≤ 0.05. Differentially expressed genes were subjected to in-depth clustering analysis and functional enrichment analysis.

### Metabolite extraction and UPLC-MS analysis

The samples were extracted using a pre-cooled methanol-water solution containing an internal standard. After grinding the tissue, applying low-temperature ultrasonication and settling, the supernatant was collected by centrifugation. Membrane filtration was then performed to prepare the analytical samples, with portions of these being pooled to create a mixed quality control (QC) sample. All samples were transferred to injection vials for analysis. Mass spectrometry data analysis was conducted using a Q Exactive mass spectrometer (Thermo Fisher Scientific, Waltham, MA, USA). Mass spectrometry data were acquired in both positive and negative ion modes. The scan ranges were set to m/z 125–1500 and 100–1500, respectively, with a primary resolution of 70,000 and a secondary resolution of 35,000. Secondary mass spectra were obtained using data-dependent acquisition (DDA, Top3) mode with multi-stage fragmentation energy settings. Chromatographic separation was performed using a Hypersil GOLD aQ Dim column (Thermo Fisher Scientific, Waltham, MA, USA) with a gradient elution of an acetonitrile–water mixture containing 0.1% formic acid as the mobile phase. The flow rate was 0.3 ml/min and the column temperature was maintained at 40 °C. Mass spectrometry raw data were imported into Compound Discoverer 3.3 software (Thermo Fisher Scientific, Waltham, MA, USA). Following analysis against online databases, the results included molecular weight, retention time, peak area, and identification information. Mean abundance and standard deviation (SD) of each metabolite were calculated for each sample group in the metabolome, and univariate analysis was performed to obtain fold changes and p-values. Differential metabolites were then identified using the criteria of a fold change ≥ 1.2 or ≤ 0.83 and a p-value < 0.05.

### Statistical analysis

All data were subjected to a one-way analysis of variance (ANOVA) using IBM SPSS Statistics 26.0, and mean differences were compared by Duncan’s multiple range test. Graphical representations were generated using Origin 2021 software. Each experimental result was derived from three independent biological replicates.

## Results

### Effects of exogenous H₂S on antioxidant enzyme activities and osmolytes in *R. soongorica* roots under salt stress

**Fig. 2:**
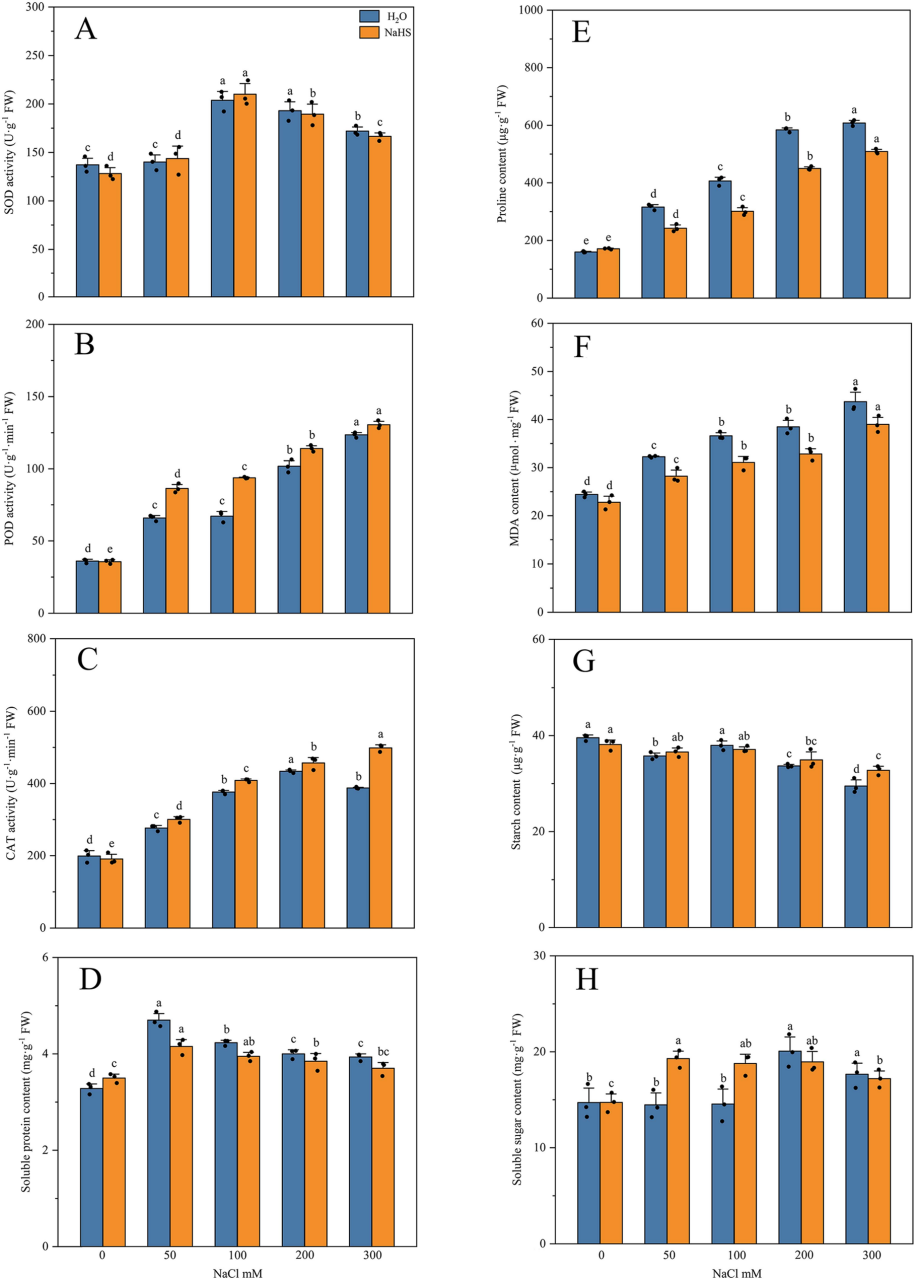
Effects of exogenous H₂S on antioxidant activities and osmoprotectant compounds in *R. soongorica* roots under salt stress. **A** SOD activity. **B** POD activity. **C** CAT activity. **D** Soluble protein content. **E** Proline content. **F** MDA content. **G** Starch content. **H** Soluble sugar content. Different letters denote significant differences obtained through Duncan’s test; error bars indicate the standard error of the mean. The origin represents the value of 3 biological replicates

As shown in Fig. 2, the activities of superoxide dismutase (SOD), peroxidase (POD), and catalase (CAT) were generally enhanced with increasing salt stress concentrations. Under WS100, SOD activity reached a maximum of 203.81 U·g⁻¹, which corresponds to a 48.50% increase compared to the control (W) (Fig. 2A). POD activity increased continuously, rising from 36.09 to 123.45 U·g⁻¹·min⁻¹ (Fig. 2B). CAT activity reached its peak under WS200 (Fig. 2C). Soluble protein content reached a peak of 4.70 mg·g⁻¹ under WS50 (Fig. 2D). Proline content increased from 159.60 µg·g⁻¹ (W) to 607.94 µg·g⁻¹ (WS300) (Fig. 2E), demonstrating that plants accumulate osmolytes to mitigate osmotic stress. Membrane lipid peroxidation was concurrently intensified, as malondialdehyde (MDA) content increased from 24.41 to 43.69 µmol·g⁻¹ (Fig. 2F), impairing cell membrane integrity. Starch content was measured to have declined from 39.57 to 29.47 µg·g⁻¹ (Fig. 2G), while soluble sugar content was significantly elevated to 20.07 mg·g⁻¹ under the WS200 treatment (Fig. 2H).

Following NaHS treatment, SOD activity reached 209.99 U·g⁻¹ under HS100, although the overall change was not significantly different from that under the corresponding single-salt stress. POD activity was significantly elevated under both HS50 and HS100 treatments compared with the corresponding single‐salt treatments. CAT activity was recorded at 498.46 U·g⁻¹ min⁻¹ under HS300. Although soluble protein content was reduced compared to the corresponding single‐salt treatment, this was accompanied by a significant increase in the activity of antioxidant enzymes (POD, CAT). This suggests that NaHS treatment reduces dependence on protein synthesis by optimizing antioxidant efficiency. Malondialdehyde (MDA) levels were consistently lower than those observed under the corresponding single‐salt treatments. This indicates that NaHS treatments had effectively inhibited membrane lipid peroxidation. Proline content was 508.73 µg·g⁻¹ under HS300 treatment, which was a 16.32% decrease compared with WS300 treatments (607.94 µg·g⁻¹). Concurrently, starch content had declined from 34.93 to 32.78 µg·g⁻¹ (6.16% reduction) under WS200 and WS300 treatments. It indicated that the degradation rate of starch was further slowed compared to the 12.50% decrease observed in the corresponding single-salt treatment. Soluble sugar content increased to 19.28 mg·g⁻¹ under HS50 treatments. These results collectively indicate that NaHS treatment not only promotes the accumulation of protective solutes such as soluble sugars when needed. It also helps to conserve long-term energy reserves (starch content) by improving metabolic efficiency and stress tolerance in response to salt stress.

### Effects of exogenous H₂S on endogenous H₂S content, ion balance, and root morphology in *R. soongorica* roots under salt stress

**Fig. 3:**
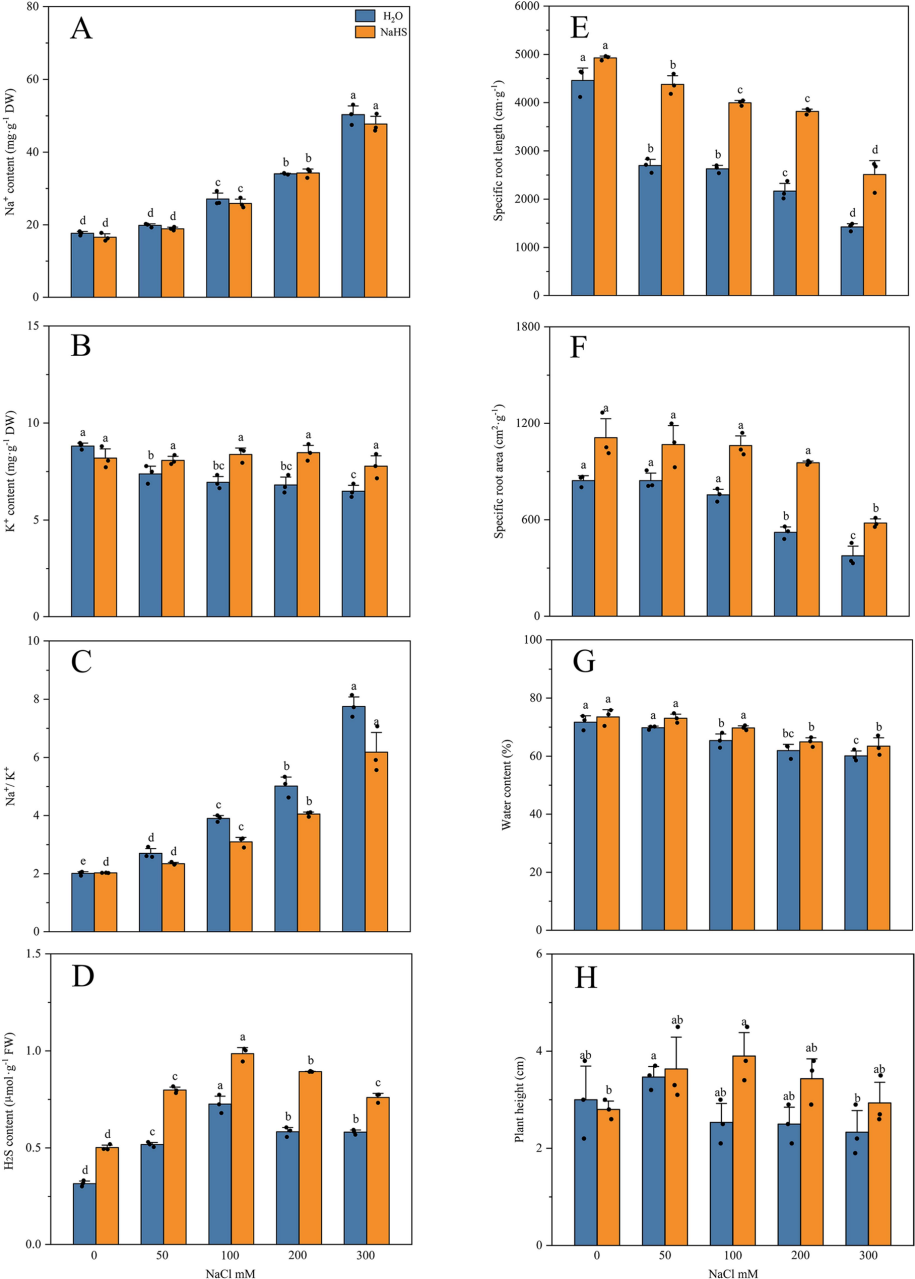
Effects of exogenous H₂S on endogenous H₂S content, ion homeostasis, and root architecture in *R. soongorica* under salt stress. **A** Na^+^ content. **B** K^+^ content. **C** Na^+^/K^+^ ratio. **D** Endogenous H₂S content. **E** Specific root length. **F** Specific root area. **G** Water content. **H** Plant height. Different letters denote significant differences obtained through Duncan’s test; error bars indicate the standard error of the mean. The origin represents the value of 3 biological replicates

As NaCl concentration increased, salt stress significantly disrupted ion homeostasis and root development in *R. soongorica* roots, leading to pronounced Na⁺ accumulation (Fig. 3A). K⁺ levels decreased from 8.81 mg·g⁻¹ to 6.49 mg·g⁻¹ (Fig. 3B), while the Na⁺/K⁺ ratio simultaneously increased from 2.01 to 7.75 (Fig. 3C). This reflects inhibited K⁺ uptake in the *R. soongorica* roots and disrupted ionic balance. Endogenous H₂S levels were also affected: H₂S content peaked at 0.73 µmol·g⁻¹ under WS100 but declined to 0.58 µmol·g⁻¹ under WS200 (Fig. 3D). Specific root length (SRL) decreased from 4,457.49 cm·g⁻¹ to 1,421.69 cm·g⁻¹ (Fig. 3E), and specific root area (SRA) decreased from 843.00 cm·g⁻¹ to 376.08 cm·g⁻¹ (Fig. 3F), consistent with inhibited root growth. These findings indicate that high salinity severely impaired *R. soongorica* root expansion. Concomitantly, this disrupted water metabolism, with root water content reduced from 71.69% to 60.10% (Fig. 3G). The growth of *R. soongorica* plant height was significantly reduced in all treatments except WS50, indicating that salt stress severely impaired the growth of *R. soongorica* (Fig. 3H).

Following NaHS treatment, plant ionic homeostasis was further optimised, and Na⁺ accumulation was markedly attenuated. Under HS300, this represented a 5.11% reduction relative to the corresponding single-salt treatment. Furthermore, K⁺ content was significantly elevated and the Na⁺/K⁺ ratio was significantly decreased compared with the single-salt treatment. This indicates that NaHS treatment facilitated K⁺ uptake while suppressing Na⁺ influx. Consequently, root growth was substantially restored. SRL increased by 76.12% under HS200 compared with the corresponding single-salt treatment. SRA was likewise significantly elevated, indicating that NaHS treatment may maintain root expansion capacity. The growth of *R. soongorica* plants height also showed a significantly elevated level compared to the corresponding single-salt treatment. Following NaHS application, the root water-retention capacity was also partially improved. Concomitant with continued NaHS treatment, endogenous H₂S levels were significantly elevated, with H₂S content at all concentrations being markedly higher than in the corresponding single-salt treatments.

### Comprehensive analysis of exogenous H₂S on physiological and biochemical indicators of *R. soongorica* under salt stress

As shown in Table S2, the salt tolerance of the plants subjected to salt stress varied non-monotonically, with scores of 0.57 and 0.59 for WS50 and HS50 treatments, respectively. Compared with WS200 (0.61), WS300(0.54), and HS300(0.46) treatment group decreased. Although the score values were similar, they represented distinct physiological responses. The low-salt treatment group received lower scores, primarily because 50 mM NaCl may only have triggered partial adjustments to osmolytes in *R. soongorica* roots. Meanwhile, proline accumulation had not been significantly enhanced, and antioxidant enzyme systems (POD and CAT) were not markedly activated. Moreover, although low NaCl concentrations are generally growth-promoting for halophytes like *R. soongorica*, the extent of positive regulation is limited. These factors suggest that physiological defence responses had not been fully mobilised under mild salt stress, resulting in relatively low salt tolerance. By contrast, treatments at moderate salt stress (100 mM NaCl) had yielded higher tolerance scores. This is because enhancements in osmolytes regulation, particularly elevated levels of proline and soluble sugars, were coupled with the full activation of the antioxidant defence system (SOD, POD, and CAT), which effectively maintains ion homeostasis. These responses reflect a highly coordinated salt tolerance mechanism in *R. soongorica*. Under high salt treatment (200 and 300 mM NaCl), excessive proline accumulation imposed metabolic stress. MDA concentrations peaked, exacerbating membrane damage. Furthermore, the compensatory capacity of the antioxidant system (SOD and CAT) was weakened. Reliance on POD alone was insufficient to counteract salt-induced damage, especially during the early stages of growth. Consequently, the integrated tolerance scores under high salt stress had fallen below those of moderate-salt treatments, indicating an imbalance in the physiological compensation of the *R. soongorica* roots by overactivation and intensified stress damage. In conclusion. Based on the analysis of salt tolerance indices on *R. soongorica* during the seedling stage under salt stress, 200 mM NaCl treatment (WS200) exerted a pronounced inhibitory effect. This was particularly evident at the initial stage, where it clearly had an inhibitory effect on the growth and development of *R. soongorica* seedlings. This concentration had previously been identified as the salt tolerance threshold for *R. soongorica* in related studies [9, 10]. Meanwhile, the most pronounced improvement in *R. soongorica* growth following NaHS application was observed under HS200 treatment (0.73), representing a 19.67% increase relative to the WS200 treatment (0.61), and approaching the score for moderate salt stress (100 mM NaCl). This enhancement may have resulted from NaHS treatment strengthening the antioxidant defence system, particularly by elevating POD activity in response to high-salt stress. Additionally, NaHS treatment optimised the regulatory compensation between osmolytes (proline and soluble sugars), enabling the roots of *R. soongorica* to exhibit enhanced salt tolerance during early growth under high-salt stress. Therefore, a 200 mM NaCl treatment was selected as the inhibitory condition for subsequent investigations into early-stage growth under salt stress at the molecular level.

Overall, the observation that both low and high salt treatments yielded lower principal component scores reflected the physiological complexity of plant responses across varying salt concentrations. Under low salt treatment, defensive mechanisms were not fully activated, whereas under high salt treatment, compensatory imbalance and aggravated morphological damage were evident. By contrast, moderate salt treatment had resulted in an optimal physiological equilibrium.

### Transcriptome sequencing analysis and functional annotation of differentially expressed genes (DEGs)

**Fig. 4:**
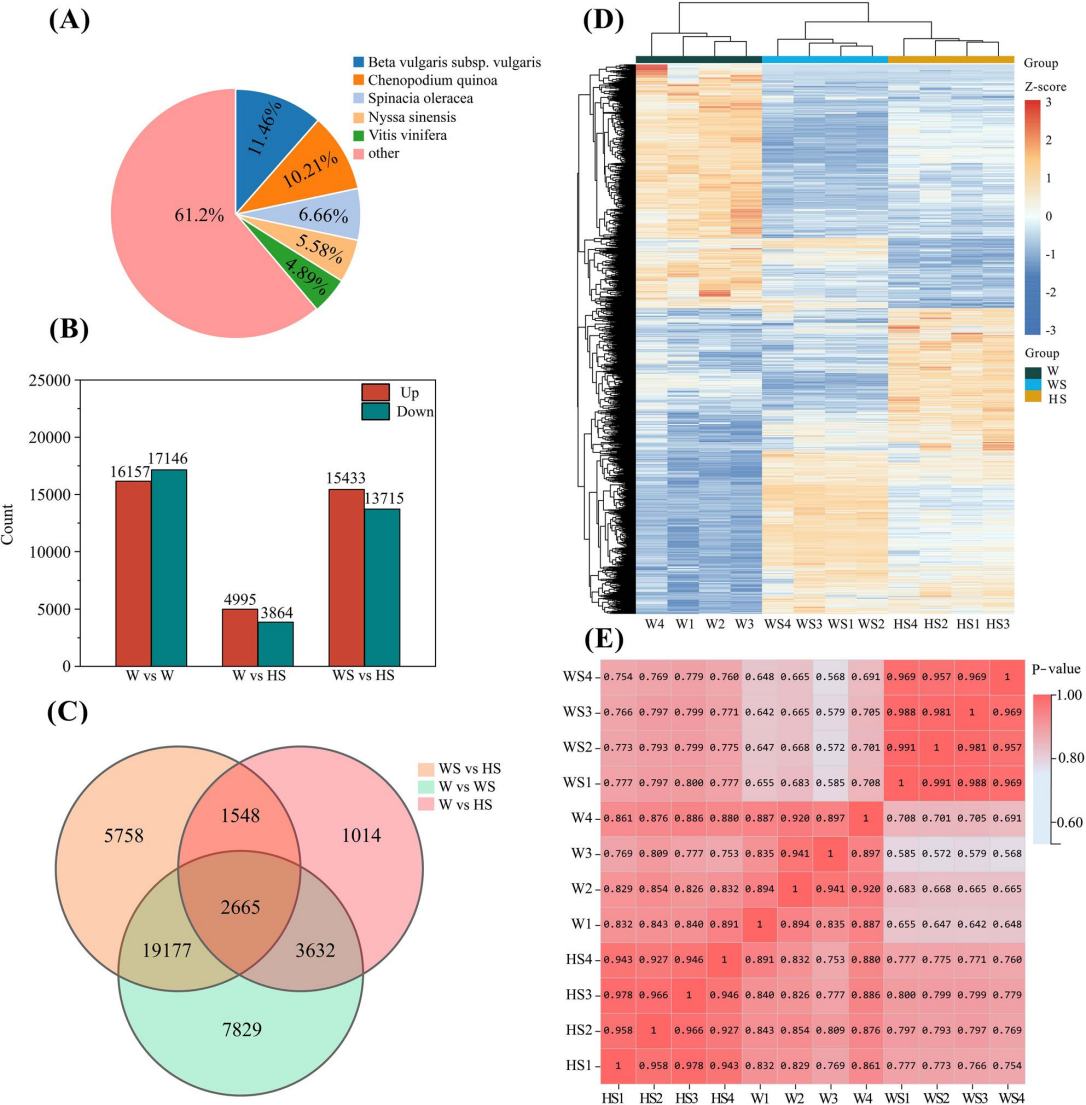
Changes in expression levels of differentially expressed genes (DEGs). **A** The detailed annotation information is based on species distribution from the non-redundant (NR) protein database. **B** Regulation of DEGs, both up and down. Up-regulation is represented by the green box and down-regulation by the red box. **C** The Venn diagram analysis of DEGs across the comparison groups. **D** The distinct expression patterns among DEGs under different treatments. **E** Heatmaps of DEGs compared between different groups

Transcriptome sequencing was performed on *R. soongorica* roots (Table S3). The three treatment concentrations were: 0 mM NaCl + H₂O (W); 200 mM NaCl + H₂O (WS); and 200 mM NaCl + 0.025 mM NaHS (HS). Each treatment comprised four biological replicates. Following RNA extraction, sequencing, and quality control, an average of 6.63 Gb of clean bases was generated per sample. The mean alignment rate to the reference gene set was 85.08%. A total of 112,872 expressed genes were detected, including 25,969 newly predicted coding genes and 86,903 non-coding genes. The mean values for Q20, Q30, and GC content were 98.83%, 96.18%, and 42.5%, respectively. This indicates high sequencing quality and suitability for further downstream analyses. Figure 4A shows that detailed annotation information is provided based on species distribution from the non-redundant (NR) protein database. Pairwise comparisons were conducted between W vs. WS, W vs. HS, and WS vs. HS, and differentially expressed genes (DEGs) were identified based on the selection criteria of |fold change| >1.42 and an adjusted p-value (Q-value) ≤ 0.05. A total of 33,303 DEGs (16,157 up-regulated and 17,146 down-regulated), 8,859 DEGs (4,995 up-regulated and 3,864 down-regulated), and 29,148 DEGs (15,433 up-regulated and 13,715 down-regulated) were detected for the respective comparison groups (Fig. 4B). Meanwhile, the Venn diagram analysis of the DEGs across the comparison groups revealed 2,665 genes that were differentially expressed in all three conditions (Fig. 4C). The expression levels across the treatment groups were standardised using Z-score transformation and hierarchical clustering heatmaps were generated to reveal the distinct expression patterns of the DEGs under the different treatments (Fig. 4D). Sample correlation heatmaps showed that the correlation coefficients among the treatment groups exceeded 0.56 (Fig. 4E).

**Fig. 5:**
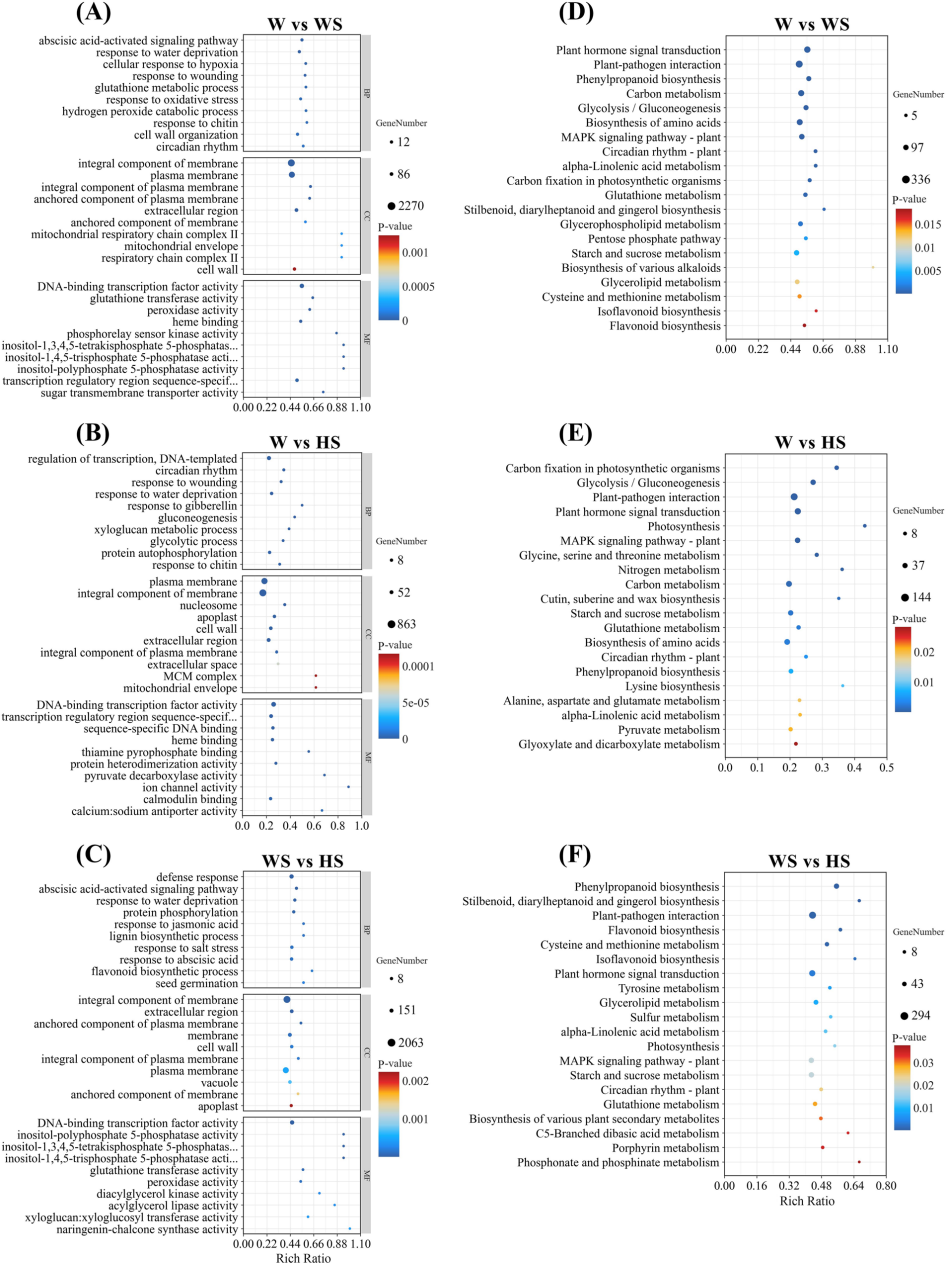
GO classification and KEGG enrichment of differentially expressed genes under different treatments. **A**-**C** GO enrichment analysis between the W vs. WS, W vs. HS, and WS vs. HS comparison groups. **D**-**F** KEGG pathway enrichment analysis between the W vs. WS, W vs. HS, and WS vs. HS comparison groups. W, WS, and HS represent 0 mM NaCl + H₂O, 200 mM NaCl + H₂O, and 200 mM NaCl + 0.025 mM NaHS treatment, respectively

Gene Ontology (GO) enrichment analysis categorised the DEGs into three distinct, interconnected groups: cellular components (CCs), biological processes (BPs), and molecular functions (MFs) (Fig. 5A-C). Within CC, terms including plasma membrane, cell wall, and integral component of membrane were significantly enriched. For BP, the most significantly enriched terms were primarily associated with metabolic processes and response to stimuli, highlighting the critical roles of cellular activity, metabolic regulation, and intercellular interactions in the response to external stress. Regarding MF, terms for binding and catalytic activity were highly represented, suggesting that the corresponding proteins primarily function in mediating biochemical reactions. KEGG pathway enrichment analysis, based on bubble plots, revealed that the DEGs identified across the three comparison groups exhibited broadly similar enrichment profiles (Fig. 5D-F). These DEGs were predominantly enriched in pathways related to plant-pathogen interaction, plant hormone signal transduction, the MAPK signaling pathway-plant, as well as glycolysis, and starch and sucrose metabolism. This finding underscores the prevailing influence of analogous pathways, including the regulation of carbohydrate utilisation, pathogen defence mechanisms and intricate signalling cascades in response to diverse treatments.

### Metabolomics analysis and functional annotation

**Fig. 6:**
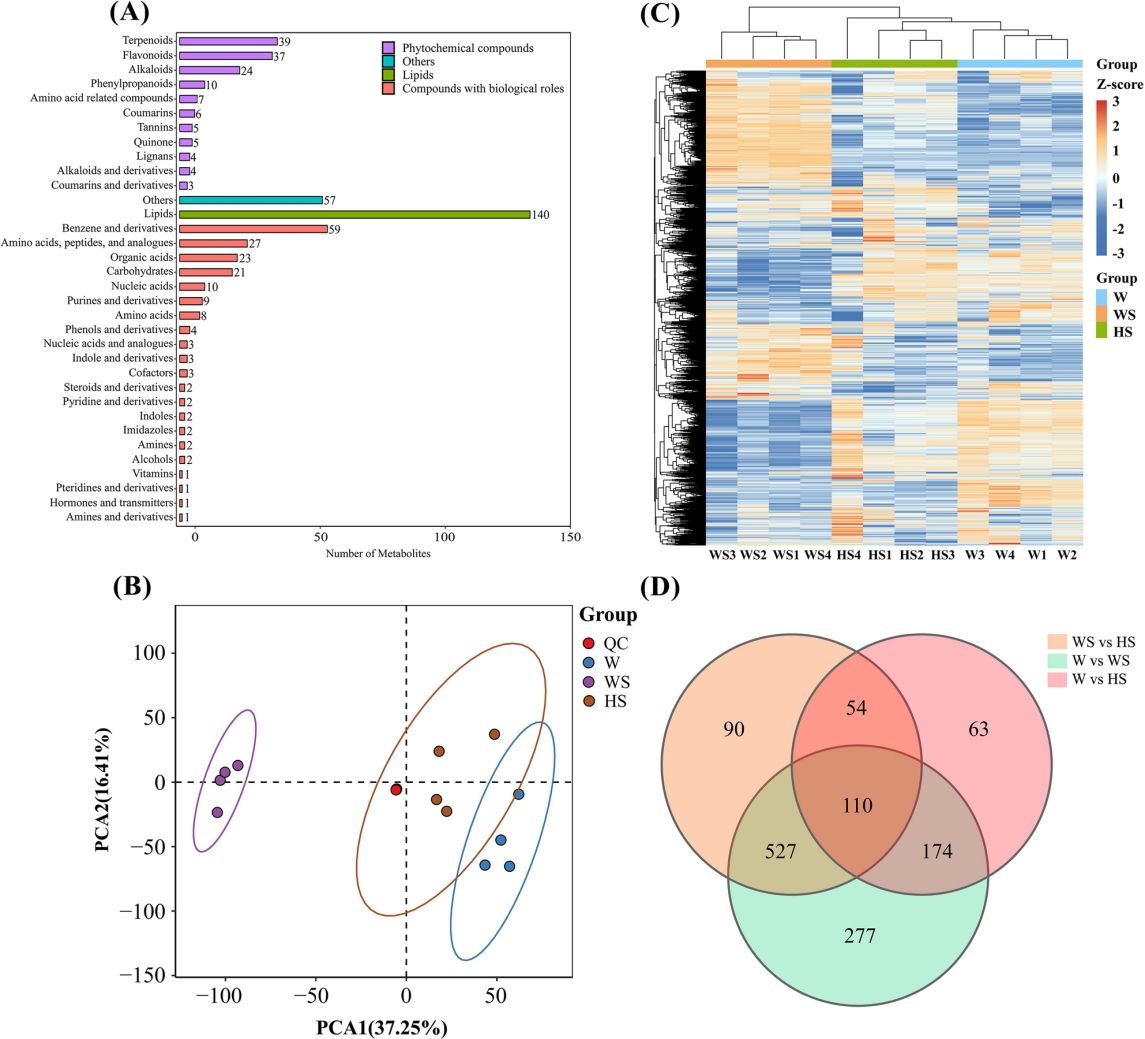
Differential analysis of metabolome expression and metabolite quality. **A** resulting in the identification of metabolites across all samples from the three treatment groups. **B** Principal component analysis (PCA) was conducted on the quantitative metabolite data from all samples. **C** Comparison of heat maps of DAMs between different treatment groups. **D** Pairwise comparison groups of DAMs on the Venn diagram

A comprehensive targeted metabolomic analysis was performed using an LC-ESI-MS/MS platform, which identified 527 metabolites in all samples from the three treatment groups (Fig. 6A). These metabolites were categorized into 34 categories (Table S4). Principal component analysis (PCA) was conducted on the quantitative metabolite data from all samples, including quality control (QC) samples. The PCA results demonstrated tight clustering of QC samples, indicating minimal system error, high experimental reproducibility, and excellent data quality (Fig. 6B). The metabolite content was then standardised, after which a hierarchical clustering heat map was constructed. This heat map demonstrated significant differences between the samples (Fig. 6C). As indicated by the screening criteria for differential accumulated metabolites (DAMs), the identification of these metabolites was based on the criteria of fold change ≥ 1.2 or ≤ 0.83 and p-value < 0.05. The distribution of DAMs among groups is illustrated in Fig. 6D. Notably, 110 DAMs were shared across all three comparison groups.

**Fig. 7:**
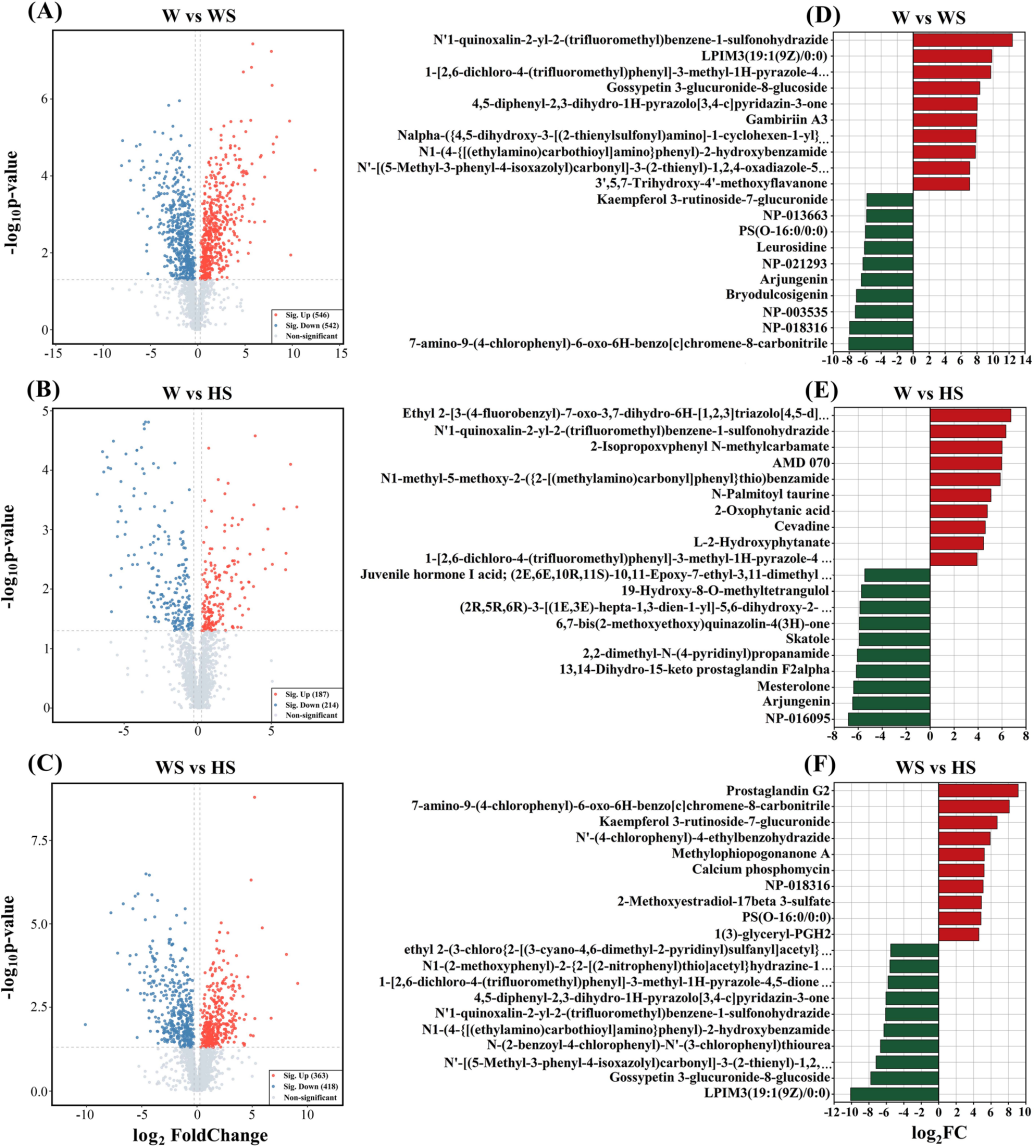
Expression analysis of different metabolites. **A**-**C** Volcano plots displaying the metabolites that are up- and down-regulated. Red points, green points, and grey points indicate the metabolites that were significantly up-regulated, down-regulated, and non-significant, respectively. **D**-**F** Top 10 up-regulated (red) and down-regulated (green) compounds

Furthermore, volcano plots were used to analyze W vs. WS, W vs. HS, and WS vs. HS comparison groups, identifying 1,088 DAMs (546 up-regulated and 542 down-regulated), 401 DAMs (187 up-regulated and 214 down-regulated), and 718 DAMs (363 up-regulated and 418 down-regulated), respectively (Fig. 7A-C). As shown in Fig. 7D, in the W vs. WS comparison group, the top ten up-regulated DAMs (red) encompassed 2 flavonoids, 1 lipid, 1 tannin, 1 amino acid-related compound, 1 phenol and derivative, and 4 other compounds. The top ten down-regulated DAMs (green) encompassed 2 terpenoids, 1 flavonoid, 1 lipid, 1 alkaloid and derivative, 1 benzene and derivative, and 4 other compounds. In the W vs. HS comparison group (Fig. 7E), the top ten up-regulated DAMs (red) encompassed 3 lipids, 3 benzene and derivatives, 1 purine and derivative, 1 phenol and derivative, 1 alkaloid and derivative, and 1 compound categorized as others. Conversely, the top ten down-regulated DAMs (green) encompassed 3 terpenoids, 2 pyridine and derivatives, 1 lipid, 1 steroid and derivative, 1 indole, 1 quinone, and 1 compound categorized as others. In the WS vs. HS comparison group (Fig. 7F), the top ten up-regulated DAMs (red) encompassed 3 lipids, 2 flavonoids, 1 coumarin and derivative, 1 benzene and derivative, 1 organic acid, 1 steroid and derivative, and 1 compound categorized as others. The top ten down-regulated DAMs (green) encompassed 3 pyridine and derivatives, 3 benzene and derivatives, 1 phenol and derivative, 1 flavonoid, 1 lipid, and 1 compound categorized as others.

### KEGG enriched pathway analysis of differential metabolites

**Fig. 8:**
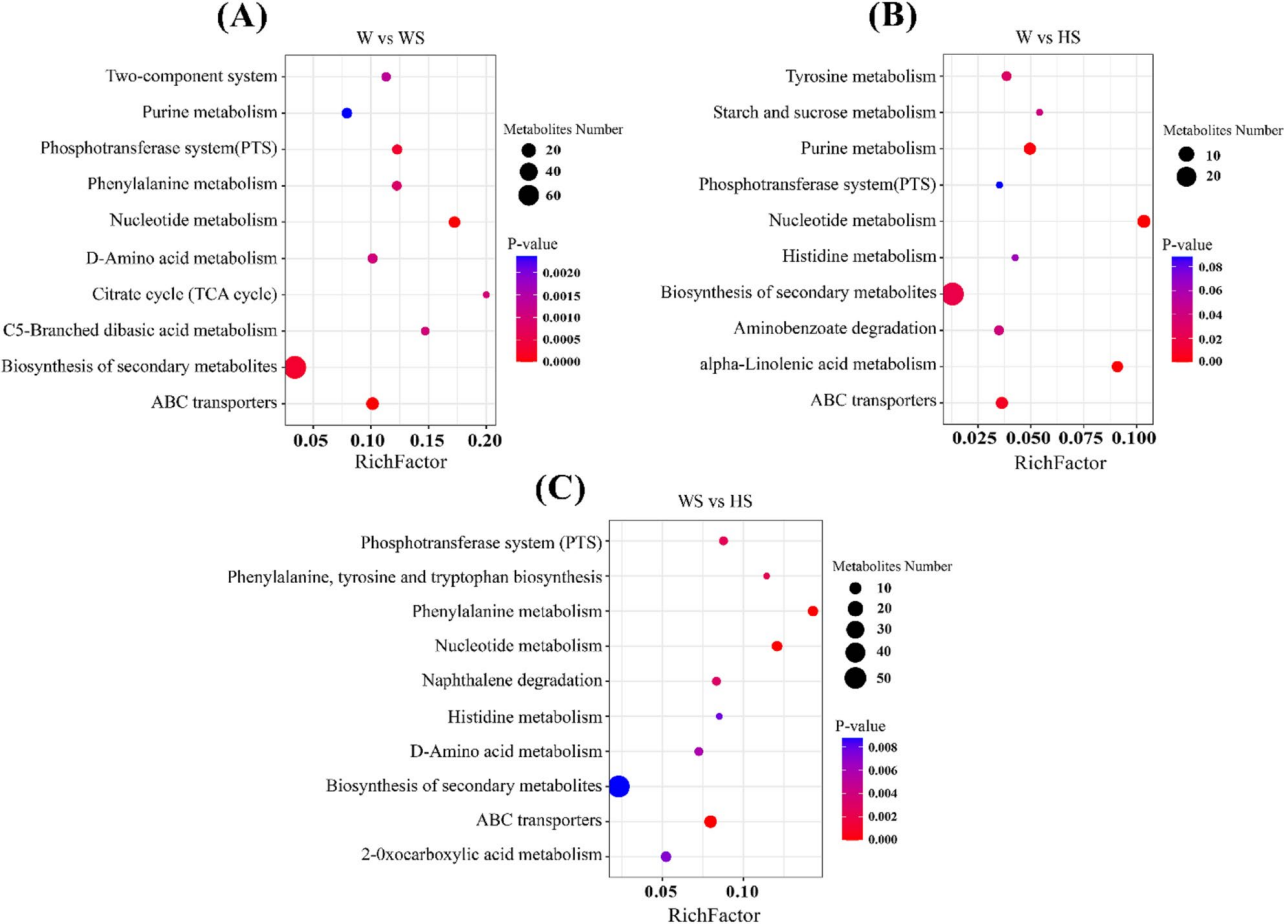
KEGG pathway enrichment analysis of differential metabolites. **A**-**C** KEGG pathway enrichment analysis of differential metabolites between the W vs. WS, W vs. HS, and WS vs. HS comparison groups. W, WS, and HS represent 0 mM NaCl + H₂O, 200 mM NaCl + H₂O, and 200 mM NaCl + 0.025 mM NaHS treatment, respectively

To identify the key metabolic pathways in *R. soongorica* roots across the three comparison groups, the top ten pathways with the highest enrichment were selected. KEGG enrichment analysis via bubble plots showed that each comparison group shared several enriched pathways (Fig. 8A-C). Among the top four pathways that were found to be significantly enriched (*P* < 0.05), the W vs. WS comparison group exhibited significant enrichment in nucleotide metabolism, ABC transporters, biosynthesis of secondary metabolites, and the phosphotransferase system (PTS). In the W vs. HS comparison group, nucleotide metabolism, α-linolenic acid metabolism, purine metabolism, and ABC transporters had been significantly enriched. Similarly, the WS vs. HS comparison group exhibited significant enrichment in phenylalanine metabolism, ABC transporters, nucleotide metabolism, and the PTS.

### Integrated analysis of transcriptomes and metabolomes

**Fig. 9:**
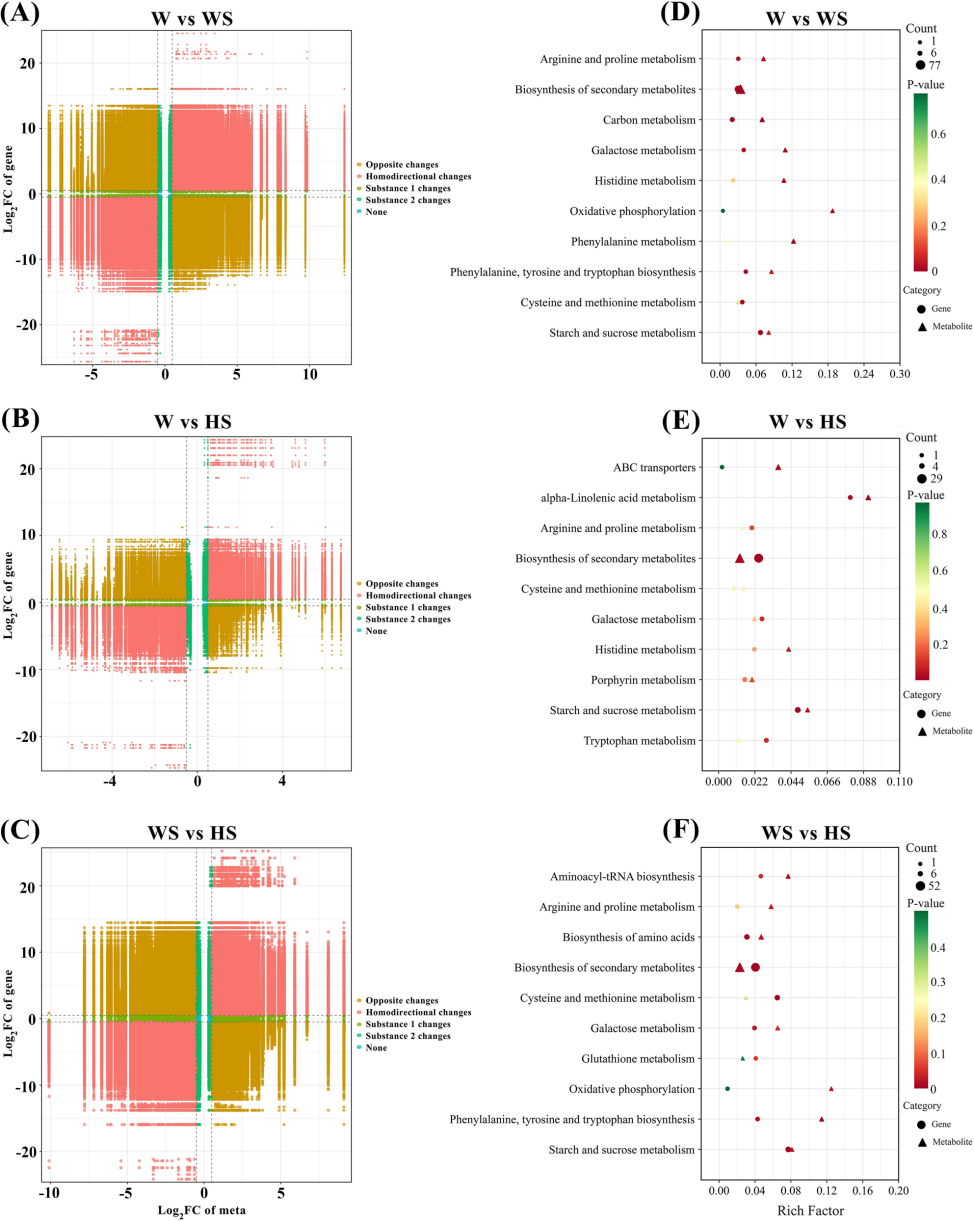
Integrated transcriptomic-metabolomic analysis and KEGG enrichment analysis. **A**-C The nine-quadrant chart visually displays multi-omics changes between different datasets. **D**-**F** KEGG enrichment analysis of shared pathways in transcriptomic and metabolomic for the W vs. WS, W vs. HS, and WS vs. HS comparison groups. W, WS, and HS represent 0 mM NaCl + H₂O, 200 mM NaCl + H₂O, and 200 mM NaCl + 0.025 mM NaHS treatment, respectively

To investigate the intricate interactions between DEGs and DAMs in *R. soongorica* roots under salt stress with exogenous H₂S application, a comprehensive co-expression network analysis was performed. Nine-quadrant plots were used to visually present multi-omics changes across the datasets (Fig. 9A-C). These analyses employed stringent thresholds (Spearman > 0.8, |Log_2_FC| >0.5), with the plots divided into nine quadrants each denoting up-regulation or down-regulation of molecules. Comparison of gene and metabolite profiles under the W vs. WS, W vs. HS, and WS vs. HS comparison groups revealed numerous genes and metabolites exhibiting concordant expression trends, thereby narrowing the scope of candidate biomarkers. Furthermore, it is important to note that metrics with highly correlated expression trends are often significant for the same phenotype or possess analogous regulatory mechanisms.

KEGG enrichment analysis was performed on the transcriptomic and metabolomic datasets (Fig. 9D-F). Five pathways were found to be consistently enriched under all treatments, revealing a striking overlap. These pathways were found to be associated with key metabolic activities, including arginine and proline metabolism, biosynthesis of secondary metabolites, cysteine and methionine metabolism, galactose metabolism, and starch and sucrose metabolism. Furthermore, histidine metabolism, oxidative phosphorylation and phenylalanine, tyrosine and tryptophan biosynthesis had been significantly co-enriched in the W vs. WS and W vs. HS comparison groups.

### Common enrichment pathway analysis

**Fig. 10:**
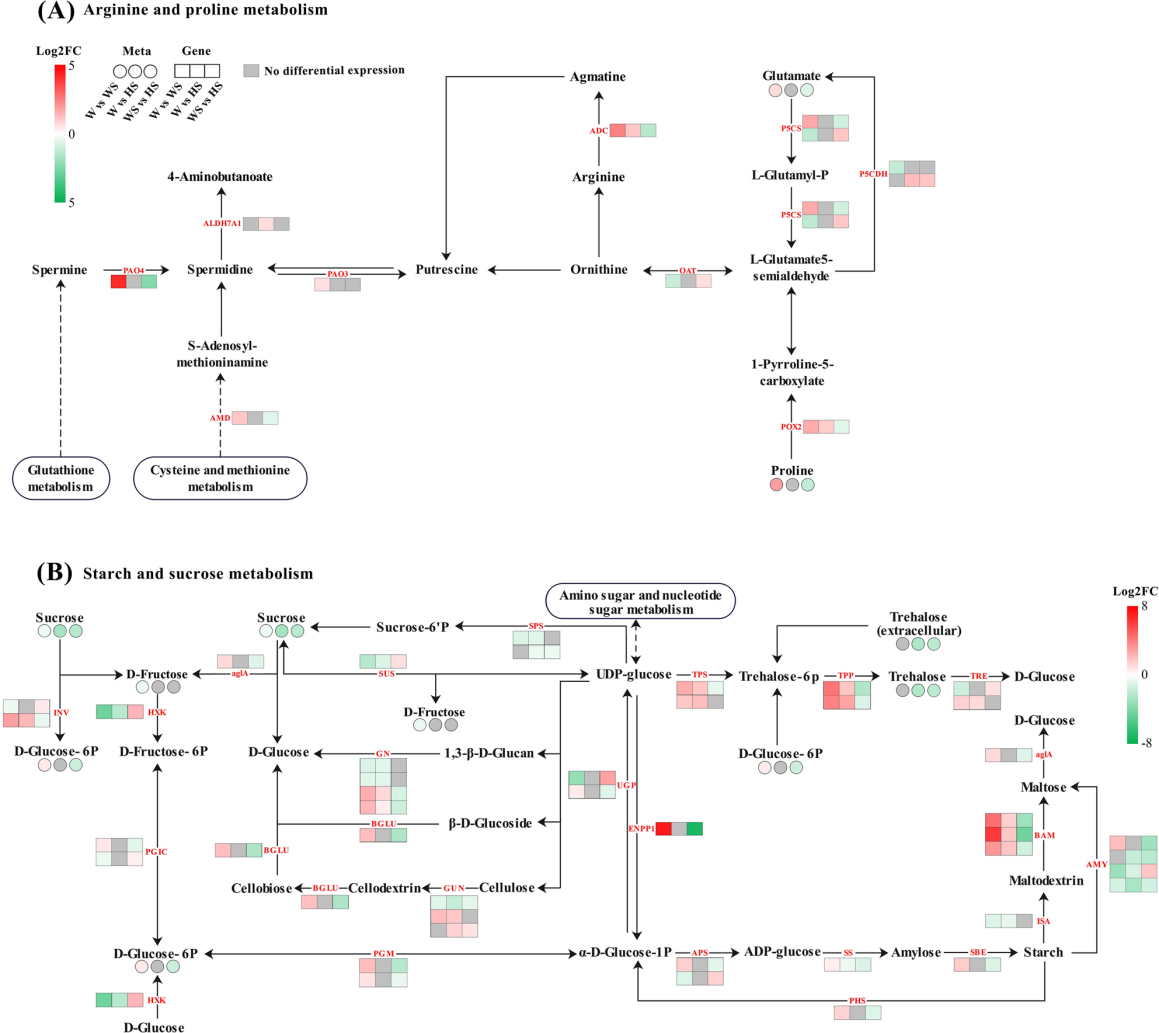
Primary metabolic pathways underlying exogenous H₂S-mediated salt stress responses in *R. soongorica* roots. The main metabolic pathways of *R. soongorica* roots under different treatments: (**A**) arginine and proline metabolism and (**B**) starch and sucrose metabolism

The integration of co-expression network analysis and KEGG enrichment analysis yielded a comprehensive understanding of the interactions between gene expression patterns and metabolite accumulation profiles. This analysis elucidated the key pathways in the R. soongorica roots in response to exogenous H_2_S under salt stress. Integrated transcriptomic and metabolomic analyses indicated that the key differential genes and metabolites were predominantly concentrated in starch and sucrose metabolism and arginine and proline metabolism (Fig. 10A-B).

In particular, the starch and sucrose metabolism pathway encompassed multiple DEGs and DAMs. The DEGs predominantly included α-glucan phosphorylase (*PHS*), sucrose synthase 2 (*SUS*), probable sucrose-phosphate synthase 1 (*SPS*), trehalose-6-phosphate synthase (*TPS*), and trehalose-6-phosphate phosphatase (*TPP*). The DAMs were mainly represented by sucrose, d-glucose-6-phosphate, d-fructose, and trehalose. Within the arginine and proline metabolism pathway, DEGs were chiefly delta-1-pyrroline-5-carboxylate synthase (*P5CS*), delta-1-pyrroline-5-carboxylate dehydrogenase (*P5CDH*), ornithine aminotransferase (*OTA*), and spermidine synthase (*SPD*), whereas DAMs were principally L-proline and L-glutamate. Although data on certain DEGs and DAMs in these pathways were unavailable, the up- and down-regulation of genes and enzymes had nonetheless driven further shifts in metabolite levels. This was primarily achieved through modulation of osmolyte accumulation and energy metabolism, in order to counteract salt stress in *R. soongorica* roots.

## Discussion

**Fig. 11:**
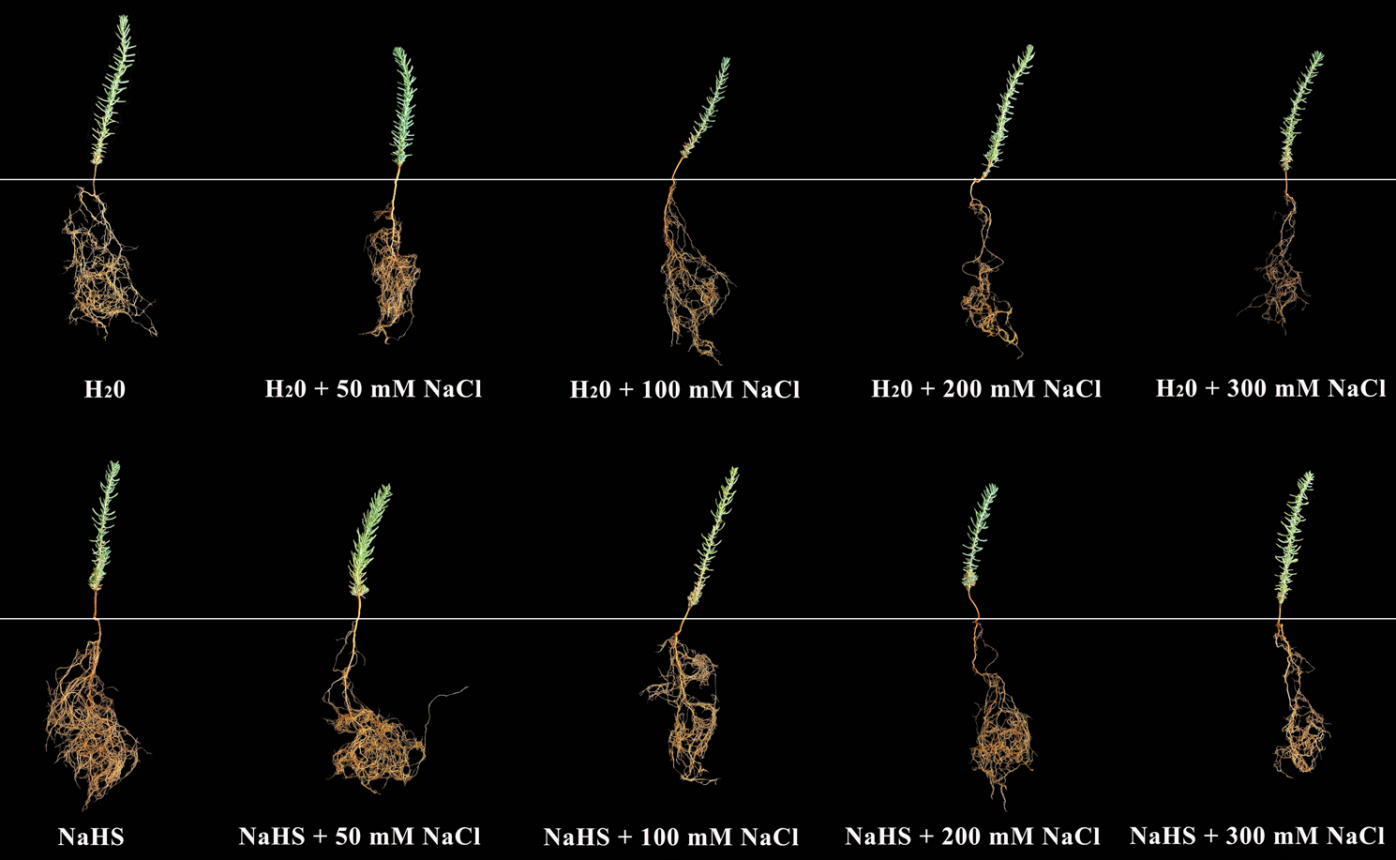
Growth status of *R. soongorica* roots under salt stress based on different treatments

Previous studies have demonstrated that exogenous H₂S influences various aspects of plant biology, including but not limited to nutrient uptake, physiological metabolism, stress tolerance, and growth-development processes [30, 31]. The results of this study, as illustrated in Fig. 1, show the growth status of *R. soongorica* after treatment with exogenous H₂S. Under salt stress, the root of plants is the initial organ to be affected at the seedling stage [47]. For *R. soongorica*, the growth of its root was inhibited and its structure was changed under high-intensity salt stress, which was consistent with its universality. The specific root length (SRL) was defined as the root length per unit root mass, whereas the specific root area (SRA) referred to the root surface area per unit root mass [48, 49]. These parameters typically declined under salt stress because it reduced root biomass, root length and root surface area. This leads to a decrease in SRL and SRA. In this study, under the WS200 treatment, the root architecture of *R. soongorica* exhibited similarly significant changes relative to the control (W) (Fig. 11). This finding is consistent with the results of most studies [50]. The problem may be that too much salt stops the growth of *R. soongorica* roots and the development of new roots. This affects the root’s ability to absorb water and nutrients [48, 49]. This was corroborated by the marked reduction in the water content of *R. soongorica* roots under the WS200 treatment. This result is similar to what many other studies on *Malus hupehensis* seedlings have found. NaHS pretreatment has been shown to significantly preserve root vigor and architecture in *M. hupehensis.* Moreover, NaHS treatment has been demonstrated to enhance salt-stress tolerance in *M. hupehensis* by regulating root sulfur‐containing compounds and cell‐wall-related gene expression [22]. Concurrently, exogenous NaHS application has been reported to facilitate root growth and development in *Nicotiana tabacum* [51].

In the transcriptomic analysis, carbon metabolism pathways were significantly enriched in the W vs. WS and W vs. HS comparison groups (Fig. 5D-E). Such pathways have been demonstrated to influence root architecture by modulating the expression of genes that regulate root growth [52, 53]. Glucose-6-phosphate 1-dehydrogenase (*G6PDH*) is not only a member of the carbon metabolism pathway, but it is also the rate-limiting enzyme of the pentose phosphate pathway (*PPP*), and its gene up-regulation indicates that plants urgently need NADPH to cope with salt stress [54, 55]. Concurrently, when metabolic processes are characterised by elevated flux and diminished inventory levels, it is indicative of a situation wherein the rate of NADPH consumption surpasses the capacity for its synthesis [55, 56]. This is indicative of the significant demand for NADPH in the root, which is attributable to oxidative stress and biosynthesis. In this study, *G6PDH* was found to be up-regulated in both the W vs. WS and W vs. HS comparison groups; whereas its direct metabolic product in metabolomics, delta-gluconic acid delta-lactone, was down-regulated only in the W vs. WS comparison group. This indicates that *R. soongorica* roots adopt a survival strategy of sacrificing energy efficiency in exchange for NADPH in response to salt stress, resulting in the diversion of energy resources to defence pathways. Although it may limit the root growth (reduced dry matter accumulation), it can increase the survival probability of *R. soongorica*. The significant reductions in root architecture indices under salt stress further substantiated this conclusion as well. And when we analyzed the changes in the number of carbon metabolism pathway genes. The results suggest that NaHS treatment resulted in most carbon metabolism-related genes tending to a silenced or basal expression state, with a significant reduction in overall pathway activity. However, NaHS treatment resulted in a significant enhancement of root growth and function (water content, specific root length, specific root area). Despite the seemingly contradictory associations. This finding may be indicative of the resource-optimisation allocation hypothesis. The NaHS treatment may drive plants to prioritize the allocation of limited carbon resources to root architecture (increasing length and surface area), something we also identified evidence for in genes whose carbon metabolism was enriched in W vs. WS and W vs. HS comparison groups as well. Among all the up-regulated genes in the two comparison groups, we identified the carbon metabolism core enzymes: pyruvate kinase (*PK*), fructose-1,6-bisphosphatase (*FBPase*), probable 6-phosphogluconolactonase 4 (*6PGL4*), catalase (*CAT*), acyl-CoA oxidase *(AOX*), and glutamate dehydrogenase B (*GDHB*) were up-regulated in the W vs. HS comparison group. Intriguingly, all of these genes had either been unexpressed or down-regulated in the W vs. WS comparison group. In the carbon metabolism pathway, these five classes of compounds have been shown to supply energy and carbon skeletons for root growth [57], maintain root growth activity [58], support membrane biogenesis and energy transduction [59], and regulate carbon-nitrogen homeostasis and osmolytes adjustment [52]. The results indicate that this is different from the strategy of sacrificing energy for survival under single-salt treatment. Carbon resource allocation in the *R. soongorica* root under salt stress was further optimized after NaHS treatment, which enhanced its stress tolerance by regulating the relevant core genes of carbon metabolic pathways, and ultimately drove the improvement of morphology and function of the *R. soongorica* root. Instead of maintaining the regulation of a wide range of carbon metabolizing enzyme activities.

Plants under salt stress reallocate energy and devote more energy and resources to synthesize osmoregulatory substances (such as proline) and antioxidant substances in response to salt damage [60]. As a principal osmolyte, proline content in plant roots had typically increased markedly under salt stress [61]. In this study, proline levels in *R. soongorica* roots had likewise risen significantly with escalating NaCl concentration. This finding is consistent with the results of most studies [62]. However, the proline content was significantly reduced by exogenous H₂S treatment compared to the single-salt treatment (Fig. 2E). The reason might be the same as some research results [63]. This response may have been due to NaHS-mediated mitigation of oxidative damage in *R. soongorica* roots under salt stress, thereby diminishing the reliance on excessive proline accumulation. In this study, for the regulation of proline in *R. soongorica* roots. We found that both arginine and proline metabolism were enriched in the W vs. WS and W vs. HS comparison groups in the transcriptome. The number of unexpressed genes was increased significantly under NaHS treatment (W vs. WS) 51 vs. (W vs. HS) 84, suggesting that NaHS treatment may inhibit the activity of genes of the arginine and proline metabolism pathway. Meanwhile, the number of up-regulated genes was further reduced (W vs. WS) 37 vs. (W vs. HS) 9, indicating that exogenous H₂S reinforced the inhibition of some genes under this pathway under salt stress. This main reason may be the result of the *R. soongorica* roots in response to different salt stress strategies. In the W vs. WS comparison groups, transcriptomic analysis revealed that *OAT* was down-regulated. Metabolomic profiling showed a concomitant decrease in γ-glutamyl-γ-aminobutyraldehyde. This pattern indicated that *OAT* repression had effectively severed the primary ornithine-to-proline synthesis pathway [64, 65]. Intriguingly, both the proline content in physiological assays and the abundance of the metabolite L-proline had nevertheless increased. The possible reason is that the proline metabolism of the *R. soongorica* roots bypasses the OAT pathway; the precursor γ-glutamyl-γ-aminobutyraldehyde is directly converted to proline via *P5CS* [66, 67]. The activation of *P5CS* and the concomitant up-regulation of l-glutamic acid abundance further corroborated this alternative pathway as well (Fig. 10A). But at the same time, the consequence is the sacrifice of energy efficiency (consumption of ATP and NADPH). This is because L-glutamic acid to L-proline requires the consumption of 2 NADPH + 1 ATP, while the energy consumption of ornithine to L-proline is only 1 NADPH [68]. This indicates that changes in the synthetic pathway under salt stress further increase the energy consumption of proline metabolism in *R. soongorica* roots compared to normal synthetic conditions.

In the W vs. HS comparison group, NaHS treatment induced up-regulation of aldehyde dehydrogenase family 7 member A1 (*ALDH7A1*). Unlike canonical aldehyde dehydrogenases, *ALDH7A1* has been shown to oxidize reactive aldehydes such as malondialdehyde (MDA) [69–71]. In this study, MDA levels in *R. soongorica* roots had risen markedly under salt stress, whereas exogenous H₂S application had effectively reduced MDA content (Fig. 2F). It also further corroborated the role of *ALDH7A1* and the reason for the reduction of MDA content [71]. Concurrently, we further found that arginine decarboxylase (*ADC*) was up-regulated in the W vs. HS comparison group, leading to the synthesis of putrescine. This process provides the substrate α-aminoadipic semialdehyde (*AASA*) for ALDH7A1 synthesis and formed a metabolic cycle [72]. Overactivation of polyamine oxidase (*PAO*) had been avoided, thereby preventing the exhaustion of putrescine and enabling sustainable stress adaptation [73]. These findings suggest that NaHS-treated *R. soongorica* roots counteract salt-induced damage through a combination of proline and *ALDH7A1*-specific activity, rather than relying solely on energetically costly proline synthesis. In terms of energy expenditure, *ALDH7A1* served as a pivotal node in H₂S-reconfigured metabolism, catalyzing the reduction of NADP⁺ to NADPH, which was then recycled by the antioxidant machinery [70]. The net energy cost of this process was substantially lower than that incurred by proline synthesis under the single-salt treatment. In conclusion, it was found that NaHS treatment remodeled the unsustainable metabolism (high-energy proline-synthesis pathway) of the *R. soongorica* roots under single-salt treatment into an energy-efficient biosynthetic pathway through the “ALDH7A1-ADC” synergistic axis, which optimized both energy balance and osmolytes protection.

Soluble sugars and starches are the main energy sources for plants when they are responding to salt stress [74]. Soluble sugars had also been important in regulating the water balance and stability of cells, which helps them to withstand salt stress [75]. Starch, on the other hand, has been used to store energy and adjust osmolality indirectly [74]. When plants are stressed by salt, they usually make more soluble sugars and less starch [61]. In this study, soluble sugar levels in *R. soongorica* roots had risen significantly with escalating salt stress, whereas starch content declined correspondingly. This result is similar to those of most other studies [61, 75]. However, starch degradation was further retarded and soluble sugar content was maintained at stable levels after NaHS treatment. This effect may have been attributable to NaHS-mediated optimization of the synergistic interactions between soluble sugars and other osmolytes, thereby alleviating salt-induced oxidative damage in *R. soongorica* roots. Moreover, the fine-tuned regulation of starch catabolism has provided a reliable and sustained energy supply for osmolyte-adjustment metabolism of the *R. soongorica* roots under salt stress [63].

At the molecular level, we identified an enrichment of multiple energy metabolism pathways involved in the regulation of soluble sugars and starch in both the transcriptome and the metabolome. Apart from the pentose phosphate pathway, which was uniquely enriched in the W vs. WS group, the following metabolic pathways were enriched across all three comparison groups: fructose and mannose metabolism, galactose metabolism, and starch and sucrose metabolism. In particular, starch and sucrose metabolism, a core pathway of energy metabolism in *R. soongorica* roots in response to salt stress, was significantly enriched in all three comparison groups. By analyzing the gene expression under this pathway, it was found that the number of unexpressed genes (W vs. WS) 167 vs. (W vs. HS) 256, the number of up-regulated genes (W vs. WS) 81 vs. (W vs. HS) 30, and the down-regulated genes of both were (W vs. WS) 73 vs. (W vs. HS) 35. It indicated that the number of expressed genes in the *R. soongorica* roots under salt stress was effectively regulated by NaHS treatment. This effect was primarily attributable to the regulation of distinct metabolic pathways by *R. soongorica* roots in response to salt-induced injury. During this response, a substantial energy supply was inevitably required, thus driving reliance on soluble sugars and starch to sustain metabolic consumption [76]. Meanwhile, we found that the starch metabolism pathway was precisely regulated by the *R. soongorica* roots under salt stress in the W vs. WS comparison group. β-Amylase (*BAM*) had been up-regulated in expression, while α-amylase (*AMY*) had exhibited a down-regulation trend. Following NaHS treatment, both *AMY* and *BAM* demonstrated similar response strategies. These results are consistent with those of most previous studies [77, 78]. *SlBAM* was differentially expressed in tomato under salt stress and was significantly up-regulated by the exogenous hormones 2,4-epibrassinolide (EBS) and α-naphthaleneacetic acid (NAA) [79]. Soaking treatment of *perilla* seeds by gibberellin (GA) resulted in significant up-regulation of genes for β-amylase and facilitated the conversion of starch to maltose [80]. However, due to interspecific diversity, both α-amylase and β-amylase genes were up-regulated in other species under salt stress [81–83]. This difference reflected the fine-tuned energy-provisioning strategy of *R. soongorica* roots. β-Amylase acts as an exonuclease, primarily targeting the non-reducing ends of starch chains. Its up-regulation promotes the process of starch catabolism, providing the necessary carbon resources and energy. In contrast, α-amylase acts as an endonuclease, and the down-regulation of its activity reduces the random breakage of starch molecules and prevents the sudden accumulation of excess reducing sugars from impacting cellular osmotic homeostasis [84, 85]. As shown in Fig. 10B, the intensity of *BAM* expression was significantly higher under single-salt treatment than under NaHS treatment, thereby markedly enhancing starch degradation rates. Consequently, the demand for starch in *R. soongorica* roots was greater under single-salt treatment than under NaHS treatment. Meanwhile, in the maltose catabolic pathway in further response to starch catabolism, the expression of alpha-glucosidase was up-regulated under single-salt treatment, whereas no expression had been detected under exogenous H₂S treatment. As a result, maltose-to-glucose conversion was more pronounced under single-salt treatment than under NaHS treatment [86], resulting in higher soluble sugar accumulation in *R. soongorica* roots subjected to single-salt treatment.

**Fig. 12:**
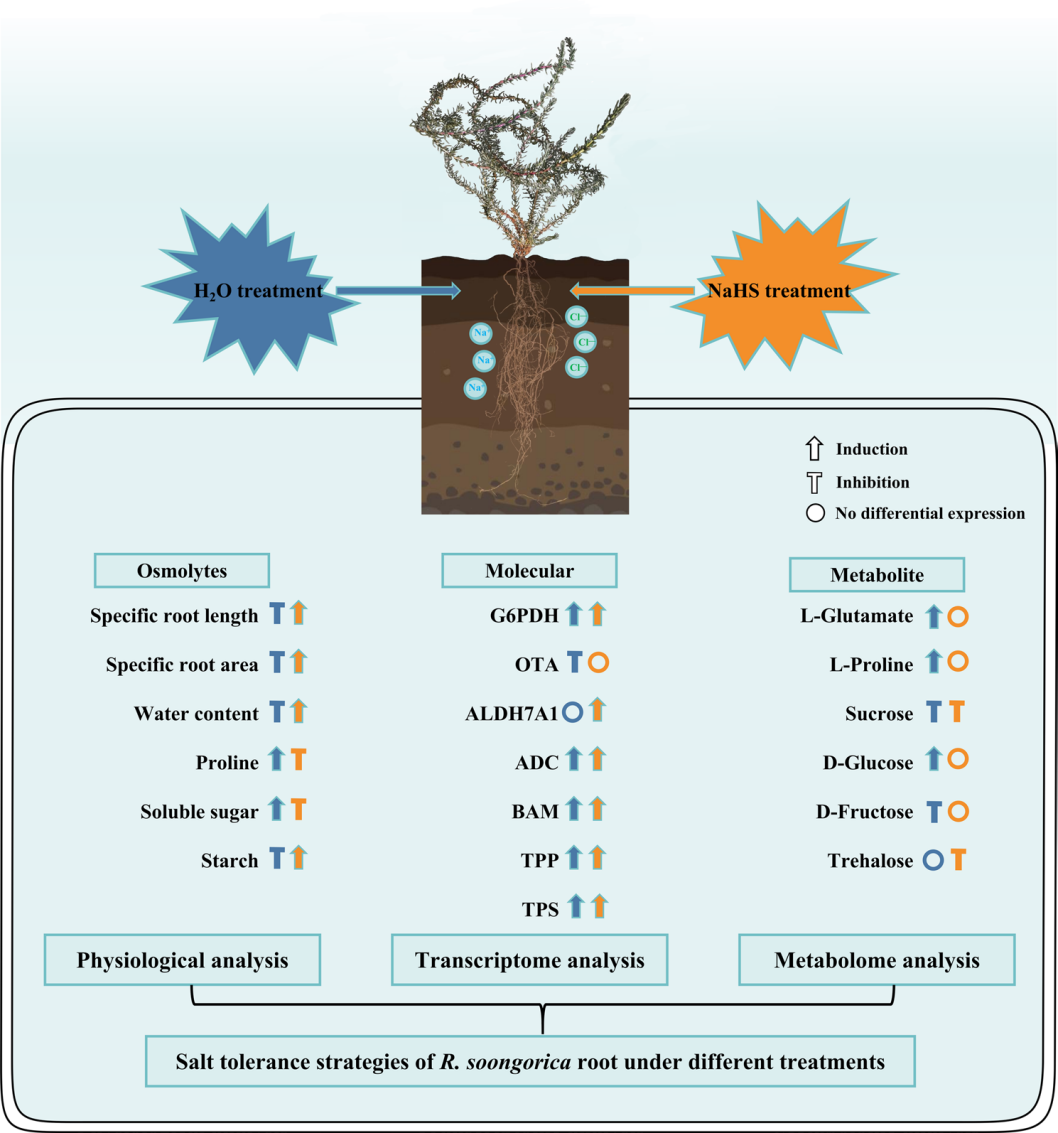
Mechanism model of salt tolerance regulation in *R. soongorica* roots under different treatments. Blue and yellow represent H₂O and NaHS treatment, respectively. Different types of symbols represent induction, repression, or no differential expression, respectively

The trehalose metabolism pathway is a central component of plant salt-stress responses, with trehalose accumulation enhancing tolerance through multiple mechanisms [87]. And it can be directly involved in osmoprotection as a compatible solute. Trehalose-6-phosphate synthase, as an essential enzyme in the biosynthesis pathway of trehalose, is responsible for catalyzing the dephosphorylation of trehalose 6-phosphate (T6-P) to produce trehalose [88]. In a high salt stress experiment in *Gracilariopsis lemaneiformis*, trehalose-6‐phosphate phosphatase 1 (*TPS1*) protein levels were elevated to 2.03‐fold those of the control plants within 48 h, accompanied by a marked increase in TPS enzymatic activity [89]. In *Medicago sativa* roots, overexpression of *MsTPS16* led to reduced reactive oxygen species accumulation and up-regulation of genes key to antioxidant‐pathway enzymes under saline‐alkaline stress [90]. Such rapid induction indicated that the trehalose biosynthetic pathway serves as a primary protective mechanism in plant salt stress responses. In this study, *TPS* showed up-regulated expression under either single-salt treatment or NaHS treatment. Interestingly, only NaHS treatment showed a down-regulation of the abundance of trehalose in the metabolome. This is likely primarily because T6-P, the precursor of trehalose, acts as a ‘central metabolic regulator’ that can coordinate plant carbon allocation and growth defense balance under stress conditions [88]. Following NaHS treatment, T6-P may be preferentially used for energy contingency (glycolysis) rather than trehalose synthesis leading to a decrease in its content or direct involvement of trehalose in osmoregulation [91] (Fig. 10B). On the one hand, it provides energy to the *R. soongorica* roots for the synthesis of osmoprotective substances. At the same time, its direct and effective osmoregulatory effect plays an energy-saving role for the sugar metabolism pathway. Through the fine-tuned modulation of starch hydrolysis and carbon partitioning, NaHS treatment achieved a dynamic balance between energy supply and osmolytes adjustment under salt stress. This further supports the resource optimization allocation hypothesis. Collectively, the growth homeostasis of the *R. soongorica* roots in response to salt stress was jointly maintained after NaHS treatment through the tertiary cascade mechanism of proline rapid response to oxidative damage, soluble sugar dominating osmolytes balance, and starch dynamic energy supply. Based on these findings, we established a conceptual model to elucidate the regulatory network mediating salt tolerance in *R. soongorica* roots under different treatments (Fig. 12). Within this framework, *R. soongorica* roots exhibited pronounced oxidative stress when exposed to high salt stress. Exogenous H₂S pretreatment induced synergistic regulation of diverse metabolic pathways, thereby effectively mitigating oxidative damage in *R. soongorica* roots.

In conclusion, this study has identified a preliminary regulatory mechanism by which exogenous H₂S enhances the salt tolerance of *R. soongorica* roots under salt stress. However, several limitations remain. The H₂S-enhanced salt tolerance regulation model developed in this study requires the core pathways and candidate genes to be functionally validated through in vivo experiments, such as gene editing. The upstream signal perception and transduction mechanisms of H₂S, along with potential post-transcriptional regulatory elements, still need to be elucidated in full using proteomics and other approaches. These follow-up studies will provide definitive evidence for the model and facilitate its translation into agricultural applications.

## Conclusion

Under salt stress, *R. soongorica* roots accumulate osmolytes and enhance antioxidant enzymatic activity, yet ionic toxicity severely impairs root growth. Exogenous NaHS application elevates endogenous H₂S, which alleviates oxidative damage and sustains root homeostasis by precisely modulating antioxidant enzymes and osmolytes. NaHS also diminishes Na⁺ accumulation and raises K⁺ concentration, thereby sustaining favorable Na⁺/K⁺ homeostasis. Multi-omics analysis revealed key osmolyte-regulating and energy metabolic pathways linked to salt stress responses. Specifically, under single-salt treatment, *R. soongorica* roots compromise energy use efficiency to prioritize NADPH production, while diverting metabolic resources toward stress defense mechanisms. Following NaHS treatment, root homeostasis is maintained via a cascade involving proline (oxidative damage), soluble sugars (osmotic balance), and starch (energy supply). This establishes dual energy and osmotic protection barriers, optimising resource allocation. These findings elucidate the role of hydrogen sulfide in the seedling establishment of *R. soongorica* under salt stress, which is crucial for breeding high-quality germplasm and for the remediation of saline-alkaline land.

## Supplementary Information

Supplementary Material 1. Table S1. Treatment gradients of exogenous H₂S application under salt stress in trays- seedling cultivation of *R. soongorica *under salt stress.

Supplementary Material 2. Table S2. Comprehensive analysis of physiological and biochemical indicators in *R. soongorica* roots under different treatments under salt stress.

Supplementary Material 3. Table S3. Statistics of transcriptomic sequencing data.

Supplementary Material 4. Table S4. Metabolite classification.

## Data Availability

The transcriptome data have been deposited at NCBI under the BioProject accession numbers of PRJNA1295811. Other data can be found in the online version of this article.
