# Supplementary material for: Integrated physiological, transcriptomic, and metabolomic analyses elucidate the mechanism of salt tolerance in Reaumuria soongorica mediated by exogenous H₂S
Source: BMC Plant Biol. 2025 Dec 1;26:29. doi: 10.1186/s12870-025-07792-0 (PMC12777452; doi:10.1186/s12870-025-07792-0)
Supplement: Supplementary file 1 — Supplementary Material 1. Table S1. Treatment gradients of exogenous H₂S application under salt stress in trays- seedling cultivation of R. soongorica under salt stress. [file 12870_2025_7792_MOESM1_ESM.docx]

**Table S1 Treatment gradients of exogenous H₂S application in trays- seedling cultivation of *Reaumuria soongorica* under salt stress.**

| **Group** | **Treatment combination** | **Treatment solution** |
| --- | --- | --- |
| NaCl + H_2_O  （Single salt treatment） | 0 mM NaCl + H_2_O（W） | H_2_O |
|  | 50 mM NaCl + H_2_O（WS50） | H_2_O |
|  | 100 mM NaCl + H_2_O（WS100） | H_2_O |
|  | 200 mM NaCl + H_2_O（WS200） | H_2_O |
|  | 300 mM NaCl + H_2_O（WS300） | H_2_O |
|  |  |  |
| NaCl + NaHS  （NaHS treatment） | 0 mM NaCl + 0.025 mM NaHS（H） | NaHS |
|  | 50 mM NaCl + 0.025 mM NaHS（HS50） | NaHS |
|  | 100 mM NaCl + 0.025 mM NaHS（HS100） | NaHS |
|  | 200 mM NaCl + 0.025 mM NaHS（HS200） | NaHS |
|  | 300 mM NaCl + 0.025 mM NaHS（HS300） | NaHS |
