# Supplementary material for: Integrated physiological, transcriptomic, and metabolomic analyses elucidate the mechanism of salt tolerance in Reaumuria soongorica mediated by exogenous H₂S
Source: BMC Plant Biol. 2025 Dec 1;26:29. doi: 10.1186/s12870-025-07792-0 (PMC12777452; doi:10.1186/s12870-025-07792-0)
Supplement: Supplementary file 2 — Supplementary Material 2. Table S2. Comprehensive analysis of physiological and biochemical indicators in R. soongorica roots under different treatments under salt stress. [file 12870_2025_7792_MOESM2_ESM.docx]

**Table S2 Comprehensive analysis of physiological and biochemical indicators in *R. soongorica* roots under different treatments under salt stress**

| Treatment | Principal component scores | | Standardization of membership function | | Synthesis score | Rank |
| --- | --- | --- | --- | --- | --- | --- |
|  | PC1 | PC2 | PC1 | PC2 |  |  |
| W | -1.36 | -1.03 | 0.00 | 0.12 | 0.07 | 5 |
| WS50 | -0.60 | 0.78 | 0.29 | 0.82 | 0.57 | 3 |
| WS100 | 0.12 | 1.26 | 0.56 | 1.00 | 0.80 | 1 |
| WS200 | 0.71 | -0.16 | 0.79 | 0.46 | 0.61 | 2 |
| WS300 | 1.13 | -0.85 | 0.95 | 0.19 | 0.54 | 4 |
|  |  |  |  |  |  |  |
| H | -1.40 | -0.95 | 0.00 | 0.17 | 0.10 | 5 |
| HS50 | -0.47 | 0.55 | 0.36 | 0.74 | 0.59 | 3 |
| HS100 | 0.08 | 1.09 | 0.56 | 0.95 | 0.79 | 1 |
| HS200 | 0.56 | 0.49 | 0.75 | 0.72 | 0.73 | 2 |
| HS300 | 1.23 | -1.17 | 1.00 | 0.09 | 0.46 | 4 |
