# Supplementary material for: Integrated physiological, transcriptomic, and metabolomic analyses elucidate the mechanism of salt tolerance in Reaumuria soongorica mediated by exogenous H₂S
Source: BMC Plant Biol. 2025 Dec 1;26:29. doi: 10.1186/s12870-025-07792-0 (PMC12777452; doi:10.1186/s12870-025-07792-0)
Supplement: Supplementary file 3 — Supplementary Material 3. Table S3. Statistics of transcriptomic sequencing data. [file 12870_2025_7792_MOESM3_ESM.docx]

**Table S3 Statistics of transcriptomic sequencing data**

| **Sample** | **Total Raw Reads (M)** | **Total Clean Reads (M)** | **Total Clean Bases (Gb)** | **Clean Reads Q20(%)** | **Clean Reads Q30(%)** | **GC(%)** |
| --- | --- | --- | --- | --- | --- | --- |
| W1 | 23.36 | 22.1 | 6.63 | 98.95 | 96.59 | 42.39 |
| W2 | 23.04 | 22.11 | 6.63 | 98.84 | 96.18 | 42.61 |
| W3 | 22.88 | 22.05 | 6.62 | 98.8 | 96.06 | 42.35 |
| W4 | 23.04 | 22.11 | 6.63 | 98.82 | 96.16 | 42.55 |
| WS1 | 23.04 | 22.03 | 6.61 | 98.78 | 96.02 | 42.54 |
| WS2 | 23.52 | 22.11 | 6.63 | 98.88 | 96.34 | 42.54 |
| WS3 | 23.36 | 22.15 | 6.64 | 98.81 | 96.1 | 42.34 |
| WS4 | 23.04 | 22.06 | 6.62 | 98.74 | 95.88 | 42.51 |
| HS1 | 23.2 | 22.1 | 6.63 | 98.82 | 96.16 | 42.66 |
| HS2 | 22.88 | 22.09 | 6.63 | 98.88 | 96.33 | 42.61 |
| HS3 | 22.88 | 22.13 | 6.64 | 98.8 | 96.06 | 42.4 |
| HS4 | 22.88 | 22.04 | 6.61 | 98.85 | 96.25 | 42.55 |
