# Supplementary material for: Integrated physiological, transcriptomic, and metabolomic analyses elucidate the mechanism of salt tolerance in Reaumuria soongorica mediated by exogenous H₂S
Source: BMC Plant Biol. 2025 Dec 1;26:29. doi: 10.1186/s12870-025-07792-0 (PMC12777452; doi:10.1186/s12870-025-07792-0)
Supplement: Supplementary file 4 — Supplementary Material 4. Table S4. Metabolite classification. [file 12870_2025_7792_MOESM4_ESM.docx]

| **Final Class** | **Count** |
| --- | --- |
| Amines and derivatives | 1 |
| Hormones and transmitters | 1 |
| Pteridines and derivatives | 1 |
| Vitamins | 1 |
| Alcohols | 2 |
| Amines | 2 |
| Imidazoles | 2 |
| Indoles | 2 |
| Pyridine and derivatives | 2 |
| Steroids and derivatives | 2 |
| Cofactors | 3 |
| Indole and derivatives | 3 |
| Nucleic acids and analogues | 3 |
| Phenols and derivatives | 4 |
| Amino acids | 8 |
| Purines and derivatives | 9 |
| Nucleic acids | 10 |
| Carbohydrates | 21 |
| Organic acids | 23 |
| Amino acids, peptides, and analogues | 27 |
| Benzene and derivatives | 59 |
| Coumarins and derivatives | 3 |
| Alkaloids and derivatives | 4 |
| Lignans | 4 |
| Quinone | 5 |
| Tannins | 5 |
| Coumarins | 6 |
| Amino acid related compounds | 7 |
| Phenylpropanoids | 10 |
| Alkaloids | 24 |
| Flavonoids | 37 |
| Terpenoids | 39 |
| Lipids | 140 |
| Others | 57 |
|  | 527 |

**Table S4 Metabolite classification**
